# Spectroscopic Constants and Anharmonic Vibrational Frequencies of C(O)OC, c-C_2_O_2_ and Their Silicon-Containing Analogues

**DOI:** 10.3390/molecules28114563

**Published:** 2023-06-05

**Authors:** Olivia A. Harwick, Ryan C. Fortenberry

**Affiliations:** Department of Chemistry and Biochemistry, University of Mississippi, University, MS 38677, USA; oaharwic@go.olemiss.edu

**Keywords:** carbon, silicon, anharmonic vibrational frequencies, rotational constants, theoretical chemistry, coupled-cluster theory, quartic force fields, comets, astrophysical ices

## Abstract

Comets are likely to contain various carbon oxide molecules potentially including C(O)OC and c-C${}_{2}$O${}_{2}$ on their surfaces and comae, as well as their silicon-substituted analogues possibly playing a role in the formation of interstellar dust grains. In this work, high-level quantum chemical data are provided to support such potential future astrophysical detection through the generation of predicted rovibrational data. Laboratory-based chemistry would also benefit from such aforementioned computational benchmarking considering these molecules’ historic computational and experimental elusiveness. Coupled-cluster singles, doubles, and perturbative triples, the F12b formalism, and the cc-pCVTZ-F12 basis set garner the rapid, yet highly trusted F12-TcCR level of theory leveraged presently. This current work points to all four molecules’ strong IR activity, coupled with large intensities, thus suggesting the potential for JWST detection. Although Si(O)OSi possesses a permanent dipole moment significantly larger than those of the other molecules of present interest, the significant abundance of the potential precursor carbon monoxide suggests that the dicarbon dioxide molecules may yet be observable in the microwave region of the electromagnetic spectrum. Thus, this present work details the likely existence and detectability of these four cyclic molecules, providing updated implications compared to previous work performed both experimentally and computationally.

## 1. Introduction

Molecules solely comprised of carbon and oxygen are more abundant than greenhouse gases or vaporous poisons. For instance, during a comet’s path around the Sun, a gaseous coma develops and is partially comprised of an assortment of molecules potentially including various carbon oxide species [1] which may even lead to the production of molecular oxygen [2,3,4]. While such chemistry has been proposed, a more complete picture of interplanetary and even interstellar cosmic carbon oxides will require further observations likely from telescopes, such as JWST [5,6,7] and the Green Bank Telescope (GBT) [1,8,9,10], each relying upon vibrational and rotational observations, respectively. However, some of the needed reference data for these observations are difficult to produce in the laboratory and could benefit from quantum chemical computations which inform observational astrochemical and experimental analyses.

One of the simplest carbon oxides beyond the typical carbon monoxide and dioxides is C${}_{2}$O${}_{2}$ which can exist as multiple isomers, including two possible cyclic forms, C(O)OC which classifies as a ketone, an epoxide, and a carbene; and the cyclic form c-C${}_{2}$O${}_{2}$ [11]. Most notably for such systems, previous computational reaction mechanisms undertaken in support of Fourier Transform Infrared (FTIR) spectroscopy-based experimental studies involving carbon oxides reveal C(O)OC as a proposed intermediate in the surface chemistry of simulated comet ices. In this previous study, linear C${}_{3}$O${}_{2}$ and carbon dioxide are shown, ultimately, to form as products due to interactions among laser-irradiated, vibrational energy pooled, condensed carbon monoxide molecules, but C(O)OC appears to be produced along the way [12]. Although shown to be likely metastable in this study [12], the singlet carbene C(O)OC could still exist long enough to be observed in the laboratory or even in astrophysical regions with modern instrumentation. The aforementioned claim is supported by 1991 GBT observations that show the related ${}^{3}\Sigma^{-}$ l-CCO is present in the Taurus Molecular Cloud (TMC-1) [13]. The question remains in relation to the C${}_{2}$O${}_{2}$ isomers. Although computation assisted experimental analysis in the reaction related to C(O)OC [12], a full vibrational or rotational characterization of this molecule is not present in the literature and would inform such studies, even if purely based in quantum chemistry. Such data could lead to confirmation of C(O)OC and would also lend credence to the observations from Ref. [12].

Both C(O)OC and c-C${}_{2}$O${}_{2}$ have been previously quantum chemically analyzed via the Hartree–Fock (HF) and MBPT(2) methods. This initial analysis implied that C(O)OC would cascade into a pair of carbon monoxide monomers, while c-C${}_{2}$O${}_{2}$ is actually not the lowest-energy structure or even lower than C(O)OC [11]. Similarly, Møller–Plesset perturbation theory (MP2) and density functional theory (DFT) computations on c-C${}_{2}$O${}_{2}$ have provided further geometry optimizations, harmonic vibrational frequencies, and zero-point vibrational energies (ZPVE) providing a spectroscopic baseline for the needed reference data of this isomer [14]. Furthermore, experiments that involved carbon- and carbon dioxide-doped helium nanodroplets coupled with DFT and coupled-cluster singles, doubles, and perturbative triples [CCSD(T)] anharmonic computations suggest that C(O)OC formation is plausible but likely unobservable [15]. Therein, the possible C(O)OC IR detection was ruled out due to the low energy of the DFT transition state leading to dissociation into its comprising pair of diatomic, heteronuclear monomers likely stemming from even minute amounts of incident photon bombardment [11,15]. As such, modern analysis for both the C(O)OC form of the carbon monoxide dimer and its c-C${}_{2}$O${}_{2}$ isomer is warranted. Establishing more reliable relative energetics, verifying geometric minima, and spectral characterization would also provide reference data for laboratory simulations and potential direct observations of comets that may include these molecules.

Additionally, the silicon analogues of these molecules [Si(O)OSi and c-Si${}_{2}$O${}_{2}$] may also be present in various astrophysical media. Silicon is known to form exceptionally strong bonds to oxygen [16], the SiO monomer has been a known interstellar molecule since 1971 from its initial detection in Sagittarius B2 [17], silicon oxides are the primary mineral component of rocky bodies and interstellar dust grains, and Si${}_{2}$O${}_{2}$ structures have shown up in reactions of silicon monoxide with water [18], possibly leading to larger silicon oxide molecular structures [19,20]. Some of the first analyses of these molecules came in 1969 when argon and nitrogen matrix-isolation IR spectroscopy assigned several c-Si${}_{2}$O${}_{2}$ IR peak assignments [21]. While debate has raged over the correct structural parameters for c-Si${}_{2}$O${}_{2}$ [21,22,23], more recent studies have provided some refined spectral characterization of these molecules [24]. Even so, the vibrational and rotational spectral datasets for these molecules are not fully elucidated for comparison to experiment or astronomical observation. Their potential role in dust formation from gas-phase molecules cannot be established until they are observed, and the present work will be providing the necessary spectral data to aid in such characterization for Si(O)OSi and c-Si${}_{2}$O${}_{2}$.

The typical means of quantum chemically computing anharmonic fundamental vibrational frequencies or zero-point averaged ($R_{\alpha }$) rotational constants is via quartic force fields (QFFs). These functions expand the internuclear Watson Hamiltonian’s potential energy via a fourth-order Taylor series and are used in astrochemical applications to support telescopic surveys [25,26,27,28,29]. This computational methodology produces spectroscopic data for individual molecules and also features good experimental agreement [30,31,32,33]. Therefore, QFF-rendered data characterizes and can ultimately aid in identifying molecules present in astrochemistry. This known quantum chemical utility will be applied to C(O)OC, c-C${}_{2}$O${}_{2}$, and their silicon analogues in order to give deeper insights into how these molecules may be observed and what such observations may imply for molecular astrophysics.

## 2. Computational Details

The QFF computations involving C(O)OC, c-C${}_{2}$O${}_{2}$, Si(O)OSi, and c-Si${}_{2}$O${}_{2}$ herein rely upon explicitly correlated CCSD(T) in the F12b formalism [34,35], the cc-pCVTZ-F12 basis set [36], core electron correlation (“cC”), and relativistic corrections (“R”). All of which define the composite level of theory utilized in this work called F12-TcCR [37] as defined below,

(1) $$E_{F12-\mathrm{TcCR}}=E_{\mathrm{CCSD}(T)-F12b/\mathrm{cc}-\mathrm{pCVTZ}-F12}+\left(E_{\mathrm{DKr}}-E_{\mathrm{DK}}\right),$$

in which “DKr” and “DK” represent the Douglas–Kroll formalism with triple-ζ relativity inclusion and exclusion, respectively [38,39]. The theoretical chemistry software package MOLPRO 2020.1 and 2022.2 facilitates *ab initio* geometry optimizations, taking core electrons into account [40,41]. Next, the C(O)OC QFF computations and their Si(O)OSi corollary involve internal-symmetry coordinate (see Equations (2)–(7)) displacements via the INTDER program [42]. Equations (8)–(13) indicate a different C(O)OC internal-symmetry coordinate definition from that described above, and this reparametrization is detailed in the Results and Discussion section. The c-C${}_{2}$O${}_{2}$ and c-Si${}_{2}$O${}_{2}$ internal-symmetry coordinates (see Equations (14)–(19) and (20)–(25), respectively) undergo displacement as well. The resultant displacements generate the 743 C(O)OC/Si(O)OSi, 353 c-C${}_{2}$O${}_{2}$, and 233 c-Si${}_{2}$O${}_{2}$ points for the single-point F12-TcCR energies. Single-point energy least-squares fitting renders the QFF. The QFF is then fit to the new energetic minimum resulting in modified force constants that INTDER alters to Cartesian coordinate format [42]. The SPECTRO program [43] reads and perturbs the aforementioned Cartesians to produce spectroscopically desired rotational constants and anharmonic vibrational frequencies from second-order perturbation theory [43,44,45,46]. These rovibrational data include $R_{\alpha }$ vibrationally averaged structures generating r${}_{0}$ geometries as well as the rovibrational data for the singly- and doubly-substituted ${}^{13}$C and ${}^{18}$O isotopologues, as well as single substitutions for ${}^{29}$Si and ${}^{30}$Si. Gaussian 16 produces B3LYP/aug-cc-pVTZ [47,48,49,50] anharmonic and double-harmonic intensities corresponding to the examined molecules’ F12-TcCR anharmonic frequencies from SPECTRO.

**Figure 1:**
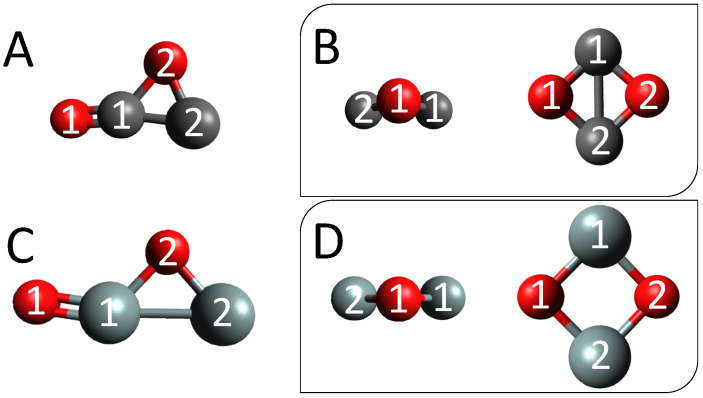
(**A**) $C_{s}$ C(O)OC top view. (**B**) $C_{2v}$ c-C${}_{2}$O${}_{2}$ side and top view. (**C**) $C_{s}$ Si(O)OSi top view. (**D**) $D_{2h}$ c-Si${}_{2}$O${}_{2}$ side and top view.

Below are the C(O)OC and equivalent Si(O)OSi ($C_{s}$) internal-symmetry coordinates; Si can replace C for the silicon analogue. See Figure 1A and C for the optimized molecular structures:

(2) $$\begin{array}{cc} & S_{1}=r\left(O_{1}-C_{1}\right) \end{array}$$

(3) $$\begin{array}{cc} & S_{2}=r\left(C_{1}-O_{2}\right) \end{array}$$

(4) $$\begin{array}{cc} & S_{3}=r\left(O_{2}-C_{2}\right) \end{array}$$

(5) $$\begin{array}{cc} & S_{4}=∠\left(O_{1}-C_{1}-O_{2}\right) \end{array}$$

(6) $$\begin{array}{cc} & S_{5}=∠\left(C_{1}-O_{2}-C_{2}\right) \end{array}$$

(7) $$\begin{array}{cc} & S_{6}=\tau \left(C_{2}-O_{2}-C_{1}-O_{1}\right). \end{array}$$

Some mixing is noted between S${}_{2}$ and S${}_{4}$ in the above coordinates for C(O)OC. As discussed in the Results and Discussion section, a new coordinate system is attempted to address potential problems:

(8) $$\begin{array}{cc} & S_{1}=r\left(O_{1}-C_{1}\right) \end{array}$$

(9) $$\begin{array}{cc} & S_{2}=\frac{1}{\sqrt{2}}\left[r\left(C_{1}-O_{2}\right)+∠\left(O_{1}-C_{1}-O_{2}\right)\right] \end{array}$$

(10) $$\begin{array}{cc} & S_{3}=r\left(O_{2}-C_{2}\right) \end{array}$$

(11) $$\begin{array}{cc} & S_{4}=\frac{1}{\sqrt{2}}\left[r\left(C_{1}-O_{2}\right)-∠\left(O_{1}-C_{1}-O_{2}\right)\right] \end{array}$$

(12) $$\begin{array}{cc} & S_{5}=∠\left(C_{1}-O_{2}-C_{2}\right) \end{array}$$

(13) $$\begin{array}{cc} & S_{6}=\tau \left(C_{2}-O_{2}-C_{1}-O_{1}\right). \end{array}$$

The c-C${}_{2}$O${}_{2}$ ($C_{2v}$; Figure 1B) internal-symmetry coordinates:

(14) $$\begin{array}{cc} & S_{1}=\frac{1}{\sqrt{2}}\left[r\left(O_{1}-O_{2}\right)+r\left(C_{1}-C_{2}\right)\right] \end{array}$$

(15) $$\begin{array}{cc} & S_{2}=\frac{1}{\sqrt{2}}\left[r\left(O_{1}-O_{2}\right)-r\left(C_{1}-C_{2}\right)\right] \end{array}$$

(16) $$\begin{array}{cc} & S_{3}=\tau \left(O_{2}-C_{2}-C_{1}-O_{1}\right) \end{array}$$

(17) $$\begin{array}{cc} & S_{4}=\frac{1}{2}\left[r\left(O_{1}-C_{1}\right)-r\left(O_{1}-C_{2}\right)-r\left(C_{1}-O_{2}\right)+r\left(C_{2}-O_{2}\right)\right] \end{array}$$

(18) $$\begin{array}{cc} & S_{5}=\frac{1}{2}\left[r\left(O_{1}-C_{1}\right)+r\left(O_{1}-C_{2}\right)-r\left(C_{1}-O_{2}\right)-r\left(C_{2}-O_{2}\right)\right] \end{array}$$

(19) $$\begin{array}{cc} & S_{6}=\frac{1}{2}\left[r\left(O_{1}-C_{1}\right)-r\left(O_{1}-C_{2}\right)+r\left(C_{1}-O_{2}\right)-r\left(C_{2}-O_{2}\right)\right]. \end{array}$$

The c-Si${}_{2}$O${}_{2}$ (*D*${}_{2h}$; Figure 1D) internal-symmetry coordinates are below and are based upon those from Ref. [26].

(20) $$\begin{array}{cc} & S_{1}=\frac{1}{\sqrt{2}}\left[r\left(O_{1}-O_{2}\right)+r\left({\mathrm{Si}}_{1}-{\mathrm{Si}}_{2}\right)\right] \end{array}$$

(21) $$\begin{array}{cc} & S_{2}=\frac{1}{\sqrt{2}}\left[r\left(O_{1}-O_{2}\right)-r\left({\mathrm{Si}}_{1}-{\mathrm{Si}}_{2}\right)\right] \end{array}$$

(22) $$\begin{array}{cc} & S_{3}=\frac{1}{2}\left[r\left(O_{1}-{\mathrm{Si}}_{1}\right)-r\left(O_{1}-{\mathrm{Si}}_{2}\right)-r\left({\mathrm{Si}}_{1}-O_{2}\right)+r\left({\mathrm{Si}}_{2}-O_{2}\right)\right] \end{array}$$

(23) $$\begin{array}{cc} & S_{4}=\tau \left(O_{2}-{\mathrm{Si}}_{2}-{\mathrm{Si}}_{1}-O_{1}\right) \end{array}$$

(24) $$\begin{array}{cc} & S_{5}=\frac{1}{2}\left[r\left(O_{1}-{\mathrm{Si}}_{1}\right)+r\left(O_{1}-{\mathrm{Si}}_{2}\right)-r\left({\mathrm{Si}}_{1}-O_{2}\right)-r\left({\mathrm{Si}}_{2}-O_{2}\right)\right] \end{array}$$

(25) $$\begin{array}{cc} & S_{6}=\frac{1}{2}\left[r\left(O_{1}-{\mathrm{Si}}_{1}\right)-r\left(O_{1}-{\mathrm{Si}}_{2}\right)+r\left({\mathrm{Si}}_{1}-O_{2}\right)-r\left({\mathrm{Si}}_{2}-O_{2}\right)\right]. \end{array}$$

All Fermi resonances and ground state dipole moment vectors are listed in the Supplementary Material (SM).

## 3. Results and Discussion

The F12-TcCR QFFs provide exceptional accuracy for the relative energies between isomers due to the inclusion of core electrons, relativity, refitting of the minimum, and anharmonic ZPVEs. This method exhibits a 1.30% mean absolute percent error for predicted anharmonic vibrational frequencies compared to those from the available experimental benchmarks [37]. As a result, C(O)OC is confidently placed 35.0 kcal mol${}^{-1}$ lower in energy than c-C${}_{2}$O${}_{2}$ in agreement with but refining previous research which computed the isomerization energy between these two structures to be 29.0 kcal mol${}^{-1}$[11]. However, the present value is computed using higher-level methods. Slight electronic and geometric differences among these species provide insight regarding the distinct aforementioned molecular energies in this present work. For instance, structurally unequal sharing of one of C(O)OC’s oxygen atoms causes an approximate 0.1 Å r${}_{0}$ difference in the two cyclic C−O bond lengths. On the other hand, the c-C${}_{2}$O${}_{2}$ carbon atoms share both oxygen atoms equally. Rationally, the c-C${}_{2}$O${}_{2}$ C−O bond length should fall within the C(O)OC cyclic C−O bond length range. The data given in the SM support this anticipated result considering the c-C${}_{2}$O${}_{2}$ r${}_{0}$ C−O bond length is about 1.39 Å, while the C(O)OC r${}_{0}$ cyclic C−O bond lengths are approximately 1.47 Å and 1.36 Å. As a final point of comparison between these isomers, the QFF fitting for c-C${}_{2}$O${}_{2}$ produces a weighted sum of squared residuals that is actually three orders of magnitude less than that of C(O)OC at $10^{-19}$ a.u.${}^{2}$ for c-C${}_{2}$O${}_{2}$ implying that the lower energy isomer has a less reliable but still tightly fitted potential surface.

Unlike the energetic comparison computed between the carbon analogues, c-Si${}_{2}$O${}_{2}$ is the lower energy isomer for the silicon-containing analogues at 55.4 kcal mol${}^{-1}$ below Si(O)OSi from the F12-TcCR refit and ZPVE-including results. This shift could affect which of these two isomers is more likely present in comets or other astrophysical environments.

### 3.1. C(O)OC

Three intensities greater than that of water’s 70 km mol${}^{-1}$ antisymmetric stretch characterize C(O)OC, 194 km mol${}^{-1}$ at 2007.0 cm${}^{-1}$ for $\nu_{1}$ (O${}_{1}$−C${}_{1}$ stretch), 79 km mol${}^{-1}$ at 1248.9 cm${}^{-1}$ for $\nu_{2}$ (O${}_{2}$−C${}_{2}$ stretch), and 103 km mol${}^{-1}$ at 310.4 cm${}^{-1}$ for $\nu_{6}$ ((C${}_{1}$−O${}_{2}$ stretch) − (O${}_{1}$−C${}_{1}$−O${}_{2}$ bend)), as indicated in Table 1.

The $\nu_{1}$ frequency at 2007.0 cm${}^{-1}$ occurs within the low-end of JWST’s NIRSpec detection limit which covers 2000 to 10,000 cm${}^{-1}$. Additionally, JWST’s MIRI instrument (350 to 2000 cm${}^{-1}$) could detect $\nu_{2}$, but $\nu_{6}$ falls just below MIRI tolerance by roughly 40 cm${}^{-1}$. However, prior work predicts the dissociation of C(O)OC into a pair of carbon monoxide molecules after overcoming a transition state of less relative energy than this fundamental frequency potentially nullifying a possible observation of the highly-intense $\nu_{1}$ fundamental [15]. However, this present study indicates that the barrier could be higher since C(O)OC exhibits two strong bonds between C${}_{1}$−O${}_{2}$ and C${}_{1}$−C${}_{2}$ which would be needed to dissociate into a pair of monomers. These bonds correspond to large force constants, F${}_{22}$ of about 3.35 mdyne/Å${}^{2}$ and F${}_{55}$ of approximately 7.39 mdyne/Å${}^{2}$, respectively. A typical C−C bond diagonal, harmonic force constant is on the order of 6−8 mdyne/Å${}^{2}$ with at least ∼3 mdyne/Å${}^{2}$ counting as covalent bonds [51,52,53,54]. This implies that these bonds are not as easily broken as the B3LYP computations from Ref. [15] would imply. As such, this intense $\nu_{1}$ may yet be observable.

In order to ensure the quality of the data produced, including this claim about the presence of C(O)OC from Ref. [15], the coordinate system is investigated further. As mentioned above, the C(O)OC QFF computation provides large mixing of internal-symmetry coordinates S${}_{2}$ (the C${}_{1}$−O${}_{2}$ stretch) and S${}_{4}$ (the O${}_{1}$−C${}_{1}$−O${}_{2}$ bend) associated with $\omega_{4}$ and $\omega_{6}$ along with their anharmonic forms, $\nu_{4}$ and $\nu_{6}$, in Table 1. Such behavior is hypothesized to be the provenance for the large relative difference between $\omega_{6}$ and $\nu_{6}$, as well as to the lesser difference among $\omega_{4}$ and $\nu_{4}$. For instance, the normal coordinate for $\omega_{4}$ is dominated by the 0.405S${}_{4}$ + 0.372S${}_{2}$ combination, while $\omega_{6}$ is 0.542S${}_{2}$− 0.374S${}_{4}$. In order to explore this possible rationalization, S${}_{2}$ and S${}_{4}$ are newly defined by the coupling of simple-internal coordinates L${}_{2}$ (the C${}_{1}$−O${}_{2}$ stretch) and L${}_{4}$ (the O${}_{1}$−C${}_{1}$−O${}_{2}$ bend) during the internal-symmetry coordinate displacement step (see description above) via INTDER [42]. This process leads to coordinate mixing rectification, but the fitting magnitudes remain the same at $10^{-16}$ a.u.${}^{2}$ for both coordinates sets. Furthermore, little to no difference in harmonic (a maximum of 0.1 cm${}^{-1}$ for $\omega_{5}$) and anharmonic (0.5 cm${}^{-1}$ for $\nu_{5}$) vibrational frequencies are shown, and the same is true for the rotational constants (0.01 MHz) comparing between the original and coupling coordinates. Hence, the large anharmonicity in $\nu_{6}$ may be genuine, and the different coordinate systems do not alter any results.

Additionally, intriguing computational Fermi resonance behavior is also present. C(O)OC’s 2$\nu_{6}=\nu_{3}$ Fermi resonance initial harmonic guess shifts significantly in the anharmonic approximation invocation. The 2$\nu_{4}$ correction dominates over the 2$\nu_{6}$ quanta regarding the $\nu_{3}$ Fermi resonance. However, explicit treatment of both 2$\nu_{6}=\nu_{3}$ and 2$\nu_{4}=\nu_{3}$ do not shift the fundamentals by more than 0.1 cm${}^{-1}$. Furthermore, low-magnitude cubic force constants (relative to the quartics) in the QFF cause a positive anharmonicity in the C(O)OC $\nu_{2}$ O${}_{2}$−C${}_{2}$ stretch using both the standard QFF approach (see Table 1), as well as the coupled S${}_{2}$ and S${}_{4}$ internal coordinates. Such positive anharmonicity has been predicted before for a similar magnitude [55,56] implying that the present results are likely physically meaningful. Unlike the promising IR data for potential JWST observation of C(O)OC, the dipole moment is small at only 0.25 D as shown in Table 2. At face value, such a small dipole implies that rotational observation of this molecule in astrophysical regions is unlikely but is still relevant to rotational spectroscopy. The differences in the rotational constants between the vibrationally-excited and ground vibrational state are given in Table 3.

Various spectral information regarding C(O)OC isotopologues is presented in the SM, but a brief standard isotopologue comparison is provided here for clarity. Heavier isotopes cause rotational constant decreases from the standard isotopologue. ${}^{12}$C(${}^{18}$O)${}^{18}$O${}^{12}$C renders the greatest percent difference (about 8.72 %) in the B${}_{0}$ rotational constant for instance. This result aligns with prediction considering that the two heavier oxygen isotopes induce the greatest change spectroscopically. Additionally, heavier isotope inclusion causes harmonic vibrational frequency decreases depending upon the motion and corresponding substitution, if any change occurs at all. Nearly all isotopologues produce expectedly decreasing anharmonic frequencies compared to the standard, but $\nu_{4}$, $\nu_{5}$, and $\nu_{6}$ become reordered in various isotopologues giving the appearance otherwise at first glance.

### 3.2. c-C${}_{2}$O${}_{2}$

Unlike the three notable C(O)OC intensities, two c-C${}_{2}$O${}_{2}$ intensities are larger than that of water’s 70 km mol${}^{-1}$ intensity, including 110 km mol${}^{-1}$ at 1024.8 cm${}^{-1}$ for the $\nu_{2}$ lateral oxygen motion and 191 km mol${}^{-1}$ at 1009.5 cm${}^{-1}$ for the $\nu_{3}$ lateral carbon motion, as described in Table 4. Although $\nu_{3}$ for c-C${}_{2}$O${}_{2}$ and $\nu_{1}$ for C(O)OC (O${}_{1}$−C${}_{1}$ stretch; Table 1) are similar in intensity, 191 km mol${}^{-1}$ and 194 km mol${}^{-1}$, respectively, the locations of these peaks render their corresponding anharmonic vibrational frequencies detectable via different JWST instruments since NIRSpec would be unavailable. Thus, unlike the $\nu_{1}$ motion in C(O)OC, $\nu_{3}$ in c-C${}_{2}$O${}_{2}$ would be observable via MIRI. Similarly, MIRI could observe the c-C${}_{2}$O${}_{2}$$\nu_{2}$ frequency. However, and in regards to the c-C${}_{2}$O${}_{2}$$\nu_{1}$ breathing motion, a positive anharmonicity is exhibited by this motion (view Table 4) in a similar fashion as that for the C(O)OC positive anharmonicity in $\nu_{2}$ (the O${}_{2}$−C${}_{2}$ stretch), but its low intensity would not be the primary feature likely observed in this range.

The 0.66 D c-C${}_{2}$O${}_{2}$ dipole moment given in Table 2 is notably larger than the C(O)OC dipole moment and is non-zero since this molecule is non-planar. Furthermore, computationally generated rotational constants aid experimental benchmarking and are given in Table 2. c-C${}_{2}$O${}_{2}$’s B and C rotational constants are several thousand MHz larger than the C(O)OC set due to the differing oxygen bonding. Notably, c-C${}_{2}$O${}_{2}$ is asymmetric, while C(O)OC is nearly prolate.

The quartic and sextic-distortion constants for c-C${}_{2}$O${}_{2}$, as well as all of the vibrational and rotational data for the ${}^{18}$O and ${}^{13}$C isotopes can be found in the SM.

### 3.3. Si(O)OSi

Although C(O)OC has three intensities larger than water’s antisymmetric 70 km mol${}^{-1}$ stretch, Si(O)OSi only has one. This 95 km mol${}^{-1}$ intensity corresponds to its $\nu_{2}$ (O${}_{2}$−Si${}_{2}$) stretching mode. This is 16 km mol${}^{-1}$ greater than that of C(O)OC for both molecules’ $\nu_{2}$. The Si(O)OSi $\nu_{2}$ (see Table 5) is 391.9 cm${}^{-1}$ smaller than that of C(O)OC (see Table 1) due to the greater mass of silicon as compared to that of carbon. Similar to the C(O)OC $\nu_{2}$, MIRI could potentially still detect transitions of this fundamental for Si(O)OSi. Unlike the positive anharmonicity associated with C(O)OC, Si(O)OSi does not offer anharmonic vibrational frequencies greater than its harmonic set.

Further differing from C(O)OC, Si(O)OSi possesses a dipole moment more than 20 times larger than C(O)OC at 5.38 D (refer to Table 2). Therefore, strong rotational activity will correspond to Si(O)OSi, as opposed to its less significant analogue. Si(O)OSi is active in the microwave region of the electromagnetic spectrum and could be observed via radio telescopes. Table 2 provides additional Si(O)OSi theoretical spectroscopic and rotational constants. However, silicon is less abundant than carbon. Furthermore, Si(O)OSi has smaller predicted B and C rotational constants (refer to Table 2) by several thousand MHz in comparison to its carbon-containing analogue (refer to Table 2). Additionally, both molecules’ κ values, Ray’s asymmetry parameter delineating whether a molecule is oblate or prolate, express only an approximate 0.06 difference, therefore, indicating their similar prolate structure. Geometric comparison among these molecules results in a 7.8${}^{\circ }$ decrease when Si${}_{1}$ replaces C${}_{1}$ in *∠*(O${}_{1}$−C${}_{1}$−O${}_{2}$), while a 23.6${}^{\circ }$ increase is caused by the substitution of Si${}_{1}$ and Si${}_{2}$ from C${}_{1}$ and C${}_{2}$ in *∠*(C${}_{1}$−O${}_{2}$−C${}_{2}$), respectively. The stronger carbon–oxygen bonding, as opposed to the silicon–oxygen, explains the above angle differences. Additionally, the Si(O)OSi QFF exhibits a good fit on the order of $10^{-16}$ a.u.${}^{2}$ like that of its carbon-containing analogue.

As with the carbon analogue, the quartic and sextic distortion constants are found in the SM along with the Si(O)OSi isotopic data.

### 3.4. c-Si${}_{2}$O${}_{2}$

Compared to the c-C${}_{2}$O${}_{2}$ results, $D_{2h}$ c-Si${}_{2}$O${}_{2}$ has two intensities larger than that corresponding to water’s 70 km mol${}^{-1}$ antisymmetric stretch. These c-Si${}_{2}$O${}_{2}$ intensities are the highly intense 496 km mol${}^{-1}$ at 814.4 cm${}^{-1}$ for the $\nu_{2}$ lateral silicon motion and 147 km mol${}^{-1}$ at 774.4 cm${}^{-1}$ for the $\nu_{3}$ oxygen atom lateral motion, as given in Table 6. The lateral oxygen motion intensity for $\nu_{3}$ c-Si${}_{2}$O${}_{2}$ is 37 km mol${}^{-1}$ greater than the c-C${}_{2}$O${}_{2}$$\nu_{2}$ intensity. However, the c-Si${}_{2}$O${}_{2}$$\nu_{3}$ lateral oxygen motion is 250.4 cm${}^{-1}$ smaller than that of c-C${}_{2}$O${}_{2}$’s $\nu_{2}$ lateral oxygen motion. Additionally, the $\nu_{2}$ lateral silicon motion intensity for c-Si${}_{2}$O${}_{2}$ is 305 km mol${}^{-1}$ greater than that of c-C${}_{2}$O${}_{2}$ for its $\nu_{3}$ lateral carbon motion. However, the $\nu_{2}$ lateral silicon motion of c-Si${}_{2}$O${}_{2}$ is 195.1 cm${}^{-1}$ less than c-C${}_{2}$O${}_{2}$’s $\nu_{3}$ lateral carbon motion. MIRI could observe both $\nu_{2}$ and $\nu_{3}$ for c-Si${}_{2}$O${}_{2}$ like it could with the carbon-containing analogue, but only these two frequencies are observable due to both symmetry and the low intensity of $\nu_{6}$. Lastly, although c-C${}_{2}$O${}_{2}$ provides a positive anharmonicity due to its $\nu_{1}$ breathing motion (see Table 4), the much smaller (∼3 cm${}^{-1}$) positive anharmonicity in c-Si${}_{2}$O${}_{2}$ originates from its $\nu_{6}$ out-of-plane bend (refer to Table 6).

Due to *D*${}_{2h}$ symmetry, c-Si${}_{2}$O${}_{2}$ has zero intensity for half of its fundamental vibrations, those which exhibit symmetry with respect to the inversion center in the irreps, unlike its *C*${}_{2v}$ carbon-containing analogue with only one null intensity for the $a_{2}$ fundamental. c-Si${}_{2}$O${}_{2}$ has a hard-zero dipole moment by symmetry in contrast to the slight out-of-plane structure inherent with c-C${}_{2}$O${}_{2}$. Hence, radio detection is not possible for this molecule. Even so, Table 2 provides spectroscopic data for c-Si${}_{2}$O${}_{2}$ if for no other reason than for comparison between analogues and also for rovibrational treatment of the rotational activity of the observable vibrationally excited states. The B and C rotational constants are several thousand MHz smaller than those of its carbon-containing analogue (refer to Table 2). Furthermore, core correlation inclusive computational exploration at the CCSD(T)-F12b/cc-pCVTZ level regarding c-Si${}_{2}$O${}_{2}$ in previous work [57] predicted B and C rotational constants that differed by 38.9 MHz and 23.5 MHz, respectively, from those of the current work. Additionally, c-Si${}_{2}$O${}_{2}$’s κ (−0.45; Table 2) is notably larger on the absolute scale than c-C${}_{2}$O${}_{2}$’s (−0.09; Table 2) suggesting the former’s more prolate form originating from the heavier mass of the silicon atoms. Structural comparison among c-Si${}_{2}$O${}_{2}$ and c-C${}_{2}$O${}_{2}$ reveal a 2.1${}^{\circ }$ increase due to silicon substitution from carbon in both (C−O−O) angles. The c-Si${}_{2}$O${}_{2}$ weighted sum of squared residuals is approximately one order of magnitude larger than its carbon-containing analogue at $10^{-18}$ a.u.${}^{2}$ for c-Si${}_{2}$O${}_{2}$, but it still produces a tight QFF fitting. Lastly, prior work [57] that involved core correlation inclusive CCSD(T)-F12b/cc-pCVTZ predicted c-Si${}_{2}$O${}_{2}$ anharmonic vibrational frequencies which differed, at most, by 2.2 cm${}^{-1}$ with those of the present work.

Full tabular information of theorized minor isotopologues of c-Si${}_{2}$O${}_{2}$ can be found in the SM. The largest difference between a more massive isotopologue and its corresponding standard originates with c-${}^{28}$Si${}^{28}$Si${}^{18}$O${}^{18}$O leading to a 36.7 cm${}^{-1}$ difference in $\omega_{1}$ and 35.7 cm${}^{-1}$ in $\nu_{1}$. The SM also contains each isotopologue’s quartic and sextic distortion constants as well.

## 4. Conclusions

The carbon oxides at the heart of this work give an indication of potentially being observable in spite of previous computational implications [15]. These new results show that not only is C(O)OC stable, this molecule is the lower energy isomer compared to c-C${}_{2}$O${}_{2}$ and has strong bonds in the three-membered ring potentially precluding simple dissociation. Hence, it may play a role in the production of C${}_{3}$O${}_{2}$ and carbon dioxide in cometary media, as theorized recently [12]. This present work has now provided the complete set of needed quantum chemically computed anharmonic vibrational frequencies and spectroscopic constants for experimental or even observational classification of C(O)OC.

Beyond this molecule, similar data are provided for c-C${}_{2}$O${}_{2}$, Si(O)OSi, and c-Si${}_{2}$O${}_{2}$. All feature motions detectable by JWST with strong IR intensities exhibited by each. However, these two carbon molecules feature meager dipole moments, while c-Si${}_{2}$O${}_{2}$ exhibits a null value due to its high symmetry. Unlike the other three, Si(O)OSi features a rather large permanent dipole moment of 5.38 D. However, even the small, but non-zero, dipole moments of C(O)OC and c-C${}_{2}$O${}_{2}$ may yet facilitate the radioastronomical observation of these forms of the carbon monoxide dimer simply due to their potential abundance in light of the highly-prevalent monomer.

Prior work [11,12,15] suggests these carbon-containing molecules are transient and/or are not true minima. Present work, however, predicts these to be minima that could also be experimentally elucidated via other reaction pathways or in different environments. Even if these molecules behave transiently, the present data provide a means of observing them in such scenarios.

## Figures and Tables

**Table 1:** C(O)OC Intensities, Frequencies, and ZPVE.

		Harmonic	Anharmonic	Harmonic	Anharmonic
	Description	*f* [km mol${}^{-1}$]	*f* [km mol${}^{-1}$]	Frequencies [cm${}^{-1}$]	Frequencies [cm${}^{-1}$]
$\omega_{1}$/$\nu_{1}$ (a${}^{\prime }$)	O${}_{1}$−C${}_{1}$ stretch	227	194	2030.4	2007.0
$\omega_{2}$/$\nu_{2}$ (a${}^{\prime }$)	O${}_{2}$−C${}_{2}$ stretch	83	79	1236.1	1248.9
$\omega_{3}$/$\nu_{3}$ (a${}^{\prime }$)	C${}_{1}$−O${}_{2}$−C${}_{2}$ bend	63	65	947.4	919.8
$\omega_{4}$/$\nu_{4}$ (a${}^{\prime }$)	(C${}_{1}$−O${}_{2}$ stretch) + (O${}_{1}$−C${}_{1}$−O${}_{2}$ bend)	50	36	518.1	454.2
$\omega_{5}$/$\nu_{5}$ (a${}^{\prime \prime }$)	out-of-plane bend	35	33	508.1	492.9
$\omega_{6}$/$\nu_{6}$ (a${}^{\prime }$)	(C${}_{1}$−O${}_{2}$ stretch) − (O${}_{1}$−C${}_{1}$−O${}_{2}$ bend)	106	103	478.3	310.4
ZPVE [cm${}^{-1}$]					2809.1

**Table 2:** C(O)OC, c-C${}_{2}$O${}_{2}$, Si(O)OSi, and c-Si${}_{2}$O${}_{2}$ Rotational Constants, Centrifugal Distortion Constants, and Dipole Moment.

	Units	C(O)OC	c-C${}_{2}$O${}_{2}$	Si(O)OSi	c-Si${}_{2}$O${}_{2}$
$A_{e}$	MHz	38,841.1	23,294.8	26,418.6	11,604.4
$B_{e}$	MHz	8384.9	15,734.3	3075.9	6087.9
$C_{e}$	MHz	6896.1	9619.4	2755.1	3992.9
$A_{0}$	MHz	38,826.2	23,060.9	26,387.8	11,547.8
$B_{0}$	MHz	8274.5	15,652.9	3062.9	6065.9
$C_{0}$	MHz	6807.2	9487.4	2742.1	3973.2
κ	–	−0.91	−0.09	−0.97	−0.45
$\Delta_{J}$	kHz	3.456	10.069	0.476	1.617
$\Delta_{K}$	kHz	151.182	−152.212	204.595	8.596
$\Delta_{JK}$	kHz	51.193	187.908	−1.266	2.145
$\delta_{J}$	Hz	682.264	3389.426	69.463	570.873
$\delta_{K}$	kHz	36.183	106.600	3.898	3.980
$\Phi_{J}$	mHz	−11.980	15.750	−0.086	1.317
$\Phi_{K}$	Hz	−2.507	7.328	3.708	0.058
$\Phi_{JK}$	mHz	−345.125	−1147.756	14.595	−8.040
$\Phi_{KJ}$	Hz	2.501	−6.033	−0.511	−0.028
$\phi_{j}$	mHz	−3.171	7.565	−0.012	0.650
$\phi_{jk}$	mHz	−257.516	−554.880	2.498	−1.502
$\phi_{k}$	Hz	3.710	1.150	0.563	0.036
Dipole	D	0.25	0.66	5.38	0.00

**Table 3:** C(O)OC, c-C${}_{2}$O${}_{2}$, Si(O)OSi, and c-Si${}_{2}$O${}_{2}$ Rotational Constant Differences [MHz].

	C(O)OC	c-C${}_{2}$O${}_{2}$	Si(O)OSi	c-Si${}_{2}$O${}_{2}$
$A_{0}$−$A_{1}$	7.9	56.4	−36.9	27.6
$B_{0}-B_{1}$	42.4	38.2	11.2	9.1
$C_{0}-C_{1}$	28.7	27.4	8.5	6.5
$A_{0}-A_{2}$	451.5	116.1	161.3	41.2
$B_{0}-B_{2}$	−23.8	10.5	0.5	2.2
$C_{0}-C_{2}$	−4.6	40.1	1.2	10.2
$A_{0}-A_{3}$	−141.4	−54.0	184.0	−19.1
$B_{0}-B_{3}$	21.8	56.6	9.0	20.2
$C_{0}-C_{3}$	14.3	30.1	13.5	11.0
$A_{0}-A_{4}$	−1327.6	−31.3	−207.0	29.6
$B_{0}-B_{4}$	56.8	36.1	8.8	15.3
$C_{0}-C_{4}$	52.2	3.0	6.4	17.0
$A_{0}-A_{5}$	−3655.6	225.6	−52.6	−3.3
$B_{0}-B_{5}$	−3.6	−41.8	−2.6	−4.6
$C_{0}-C_{5}$	−11.4	25.1	1.8	2.3
$A_{0}-A_{6}$	4694.9	155.4	12.7	37.6
$B_{0}-B_{6}$	127.6	63.3	−0.9	1.5
$C_{0}-C_{6}$	98.3	138.2	−5.5	−7.3

**Table 4:** c-C${}_{2}$O${}_{2}$ Intensities, Frequencies, and ZPVE.

		Harmonic	Anharmonic	Harmonic	Anharmonic
	Description	*f* [km mol${}^{-1}$]	*f* [km mol${}^{-1}$]	Frequencies [cm${}^{-1}$]	Frequencies [cm${}^{-1}$]
$\omega_{1}$/$\nu_{1}$ (a${}_{1}$)	(O${}_{1}$−O${}_{2}$ stretch) + (C${}_{1}$−C${}_{2}$ stretch)	14	11	1237.1	1248.4
$\omega_{2}$/$\nu_{2}$ (b${}_{1}$)	(O${}_{1}$−C${}_{1}$ stretch) + (O${}_{1}$−C${}_{2}$ stretch) − (C${}_{1}$−O${}_{2}$ stretch) − (C${}_{2}$−O${}_{2}$ stretch)	131	110	1090.7	1024.8
$\omega_{3}$/$\nu_{3}$ (b${}_{2}$)	(O${}_{1}$−C${}_{1}$ stretch) − (O${}_{1}$−C${}_{2}$ stretch) + (C${}_{1}$−O${}_{2}$ stretch) − (C${}_{2}$−O${}_{2}$ stretch)	199	191	1054.9	1009.5
$\omega_{4}$/$\nu_{4}$ (a${}_{1}$)	(O${}_{1}$−O${}_{2}$ stretch) − (C${}_{1}$−C${}_{2}$ stretch)	3	3	1019.7	999.4
$\omega_{5}$/$\nu_{5}$ (a${}_{1}$)	out-of-plane bend	7	10	444.3	369.2
$\omega_{6}$/$\nu_{6}$ (a${}_{2}$)	(O${}_{1}$−C${}_{1}$ stretch) − (O${}_{1}$−C${}_{2}$ stretch) − (C${}_{1}$−O${}_{2}$ stretch) + (C${}_{2}$−O${}_{2}$ stretch)	0	0	374.3	313.9
ZPVE [cm${}^{-1}$]					2570.9

**Table 5:** Si(O)OSi Intensities, Frequencies, and ZPVE.

		Harmonic	Anharmonic	Harmonic	Anharmonic
	Description	*f* [km mol${}^{-1}$]	*f* [km mol${}^{-1}$]	Frequencies [cm${}^{-1}$]	Frequencies [cm${}^{-1}$]
$\omega_{1}$/$\nu_{1}$ (a${}^{\prime }$)	O${}_{1}$−Si${}_{1}$ stretch	65	61	1247.0	1234.5
$\omega_{2}$/$\nu_{2}$ (a${}^{\prime }$)	O${}_{2}$−Si${}_{2}$ stretch	104	95	859.5	857.0
$\omega_{3}$/$\nu_{3}$ (a${}^{\prime }$)	Si${}_{1}$−O${}_{2}$ stretch	32	35	604.7	580.9
$\omega_{4}$/$\nu_{4}$ (a${}^{\prime }$)	Si${}_{1}$−O${}_{2}$−Si${}_{2}$ bend	34	33	443.8	435.5
$\omega_{5}$/$\nu_{5}$ (a${}^{\prime }$)	O${}_{1}$−Si${}_{1}$−O${}_{2}$ bend	22	22	207.5	204.5
$\omega_{6}$/$\nu_{6}$ (a${}^{\prime \prime }$)	out-of-plane bend	13	13	167.0	166.4
ZPVE [cm${}^{-1}$]					1755.6

**Table 6:** c-Si${}_{2}$O${}_{2}$ Intensities, Frequencies, and ZPVE.

		Harmonic	Anharmonic	Harmonic	Anharmonic
	Description	*f* [km mol${}^{-1}$]	*f* [km mol${}^{-1}$]	Frequencies [cm${}^{-1}$]	Frequencies [cm${}^{-1}$]
$\omega_{1}$/$\nu_{1}$ (a${}_{g}$)	(O${}_{1}$−O${}_{2}$ stretch) + (Si${}_{1}$−Si${}_{2}$ stretch)	0	0	861.4	847.4
$\omega_{2}$/$\nu_{2}$ (b${}_{1u}$)	(O${}_{1}$−Si${}_{1}$ stretch) − (O${}_{1}$−Si${}_{2}$ stretch) + (Si${}_{1}$−O${}_{2}$ stretch) − (Si${}_{2}$−O${}_{2}$ stretch)	511	496	828.1	814.4
$\omega_{3}$/$\nu_{3}$ (b${}_{2u}$)	(O${}_{1}$−Si${}_{1}$ stretch) + (O${}_{1}$−Si${}_{2}$ stretch) − (Si${}_{1}$−O${}_{2}$ stretch) − (Si${}_{2}$−O${}_{2}$ stretch)	149	147	785.7	774.4
$\omega_{4}$/$\nu_{4}$ (b${}_{3g}$)	(O${}_{1}$−Si${}_{1}$ stretch) − (O${}_{1}$−Si${}_{2}$ stretch) − (Si${}_{1}$−O${}_{2}$ stretch) + (Si${}_{2}$−O${}_{2}$ stretch)	0	0	578.1	564.3
$\omega_{5}$/$\nu_{5}$ (a${}_{g}$)	(O${}_{1}$−O${}_{2}$ stretch) − (Si${}_{1}$−Si${}_{2}$ stretch)	0	0	561.2	558.1
$\omega_{6}$/$\nu_{6}$ (b${}_{3u}$)	out-of-plane bend	1	1	237.4	240.8
ZPVE [cm${}^{-1}$]					1920.7

## Data Availability

All necessary data are available in the article or Supplementary Material.

## Supplementary Materials

The following supporting information can be downloaded at: https://www.mdpi.com/article/10.3390/molecules28114563/s1, including the isotopologue information, the quartic and sextic distortion constants, the force constants, geometries, Cartesian coordinates along with dipole moment vectors, and Fermi resonances for all molecules examined in this work.

## Abbreviations

The following abbreviations are used in this manuscript:

JWST	James Webb Space Telescope
GBT	Green Bank Telescope
FTIR	Fourier Transform Infrared Spectroscopy
TMC-1	Taurus Molecular Cloud
HF	Hartree–Fock
MP2	Møller–Plesset Perturbation Theory
DFT	Density Functional Theory
ZPVE	Zero-point Vibrational Energy
CCSD(T)	Coupled-cluster Singles, Doubles, and Perturbative Triples
IR	Infrared
cC	Core Electron Correlation
R	Relativistic Corrections
DK	Douglas–Kroll relativity formalism
