# Supplementary material for: Spectroscopic Constants and Anharmonic Vibrational Frequencies of C(O)OC, c-C2O2 and Their Silicon-Containing Analogues
Source: Molecules. 2023 Jun 5;28(11):4563. doi: 10.3390/molecules28114563 (PMC10254651; doi:10.3390/molecules28114563)
Supplement: Supplementary file 1 [file molecules-28-04563-s001.zip › molecules-2420354-supplementary.pdf]

Supplementary Information: Spectroscopic Constants and Anharmonic Vibrational Frequencies of C(O)OC, c-C_2O_2, and their Silicon-containing Analogues

Olivia A. Harwick and Ryan C. Fortenberry

May 31, 2023

C(O)OC dipole moment vector [a.u.]: 0.08929742, 0.03992090, 0.00000000. C(O)OC Cartesian coordinates [Bohr/a.u.]: O 0.000000000 0.059284581 -2.736156302; C 0.000000000 -0.347609661 -0.559243623; O 0.000000000 1.208339210 1.666460193; C 0.000000000 -1.340944172 1.984145457.

c-C_2O_2 dipole moment vector [a.u.]: 0.00000000, 0.00000000, 0.25822114. c-C_2O_2 Cartesian coordinates [Bohr/a.u.]: C -1.770561866 0.000000000 -0.231023337; C 1.770561866 0.000000000 -0.231023337; O 0.000000000 -1.874251686 0.173432835; O 0.000000000 1.874251686 0.173432835.

Si(O)OSi dipole moment vector [a.u.]: 2.05693724, -0.50201531, 0.00000000. Si(O)OSi Cartesian coordinates [Bohr/a.u.]: O 0.000000000 -0.038679051 -4.096222913; Si 0.000000000 -0.460667397 -1.265428612; O 0.000000000 1.829763565 0.985144156; Si 0.000000000 -0.559655459 3.037709450.

Table S1: C(O)OC Force Constants with motion coupling

$F_{1,1,0,0}$	15.023847793216
$F_{2,1,0,0}$	1.118790196621
$F_{2,2,0,0}$	2.796499259802
$F_{3,1,0,0}$	-0.096128921600
$F_{3,2,0,0}$	1.427517426504
$F_{3,3,0,0}$	5.871740163757
$F_{4,1,0,0}$	0.949357578032
$F_{4,2,0,0}$	1.120215060243
$F_{4,3,0,0}$	1.662271777467
$F_{4,4,0,0}$	1.656343785237
$F_{5,1,0,0}$	0.040760175155
$F_{5,2,0,0}$	2.531258112172
$F_{5,3,0,0}$	2.891526656896
$F_{5,4,0,0}$	2.124234146695
$F_{5,5,0,0}$	7.385054275053
$F_{6,6,0,0}$	0.267220080154
$F_{1,1,1,0}$	-106.029760641484
$F_{2,1,1,0}$	-3.040759320279
$F_{2,2,1,0}$	-2.128660304150
$F_{2,2,2,0}$	-10.882729611467
$F_{3,1,1,0}$	0.031884512560
$F_{3,2,1,0}$	-0.312812062132
$F_{3,2,2,0}$	-2.144327759126
$F_{3,3,1,0}$	0.472610710267
$F_{3,3,2,0}$	-3.589281654061
$F_{3,3,3,0}$	-34.382922408200
$F_{4,1,1,0}$	-2.784991403758
$F_{4,2,1,0}$	0.390207857911
$F_{4,2,2,0}$	-8.170465368670
$F_{4,3,1,0}$	-0.548170413529
$F_{4,3,2,0}$	-1.432345814775
$F_{4,3,3,0}$	-4.373503263910
$F_{4,4,1,0}$	-0.550252986838
$F_{4,4,2,0}$	-9.292976608105
$F_{4,4,3,0}$	0.074673012267
$F_{4,4,4,0}$	-11.938983888606

$F_{5,1,1,0}$	-0.405995660061
$F_{5,2,1,0}$	-1.222927625065
$F_{5,2,2,0}$	-2.869772028460
$F_{5,3,1,0}$	1.900335201178
$F_{5,3,2,0}$	-6.236264793530
$F_{5,3,3,0}$	-4.927692327901
$F_{5,4,1,0}$	-2.092722987053
$F_{5,4,2,0}$	-3.454855357075
$F_{5,4,3,0}$	-4.176315342550
$F_{5,4,4,0}$	-5.868797094476
$F_{5,5,1,0}$	-1.092262224457
$F_{5,5,2,0}$	-13.413277348064
$F_{5,5,3,0}$	-15.017969701043
$F_{5,5,4,0}$	-14.669145523104
$F_{5,5,5,0}$	-56.717523023035
$F_{6,6,1,0}$	-0.399076558296
$F_{6,6,2,0}$	-0.095405296434
$F_{6,6,3,0}$	-0.054881998380
$F_{6,6,4,0}$	-0.036655563606
$F_{6,6,5,0}$	-0.107957579665
$F_{1,1,1,1}$	613.522956496453
$F_{2,1,1,1}$	5.561914075058
$F_{2,2,1,1}$	4.771374752451
$F_{2,2,2,1}$	6.539270557335
$F_{2,2,2,2}$	34.182255274310
$F_{3,1,1,1}$	0.002955033735
$F_{3,2,1,1}$	1.511356544451
$F_{3,2,2,1}$	0.300599325294
$F_{3,2,2,2}$	3.410306535058
$F_{3,3,1,1}$	-2.184111870151
$F_{3,3,2,1}$	-0.599518547595
$F_{3,3,2,2}$	4.797882704805
$F_{3,3,3,1}$	2.367717959661
$F_{3,3,3,2}$	4.995458956744
$F_{3,3,3,3}$	195.717236042012
$F_{4,1,1,1}$	6.363841289446
$F_{4,2,1,1}$	1.956807810218
$F_{4,2,2,1}$	-1.005186164552
$F_{4,2,2,2}$	27.414577328014
$F_{4,3,1,1}$	2.102787490366
$F_{4,3,2,1}$	0.027466826447
$F_{4,3,2,2}$	4.802448694572
$F_{4,3,3,1}$	-0.536845288526
$F_{4,3,3,2}$	2.268647512143
$F_{4,3,3,3}$	10.586538505701
$F_{4,4,1,1}$	1.409145130466
$F_{4,4,2,1}$	-1.509524694200
$F_{4,4,2,2}$	30.051858624160
$F_{4,4,3,1}$	0.180591416890
$F_{4,4,3,2}$	7.172461539818
$F_{4,4,3,3}$	-4.621059560348
$F_{4,4,4,1}$	1.826298876741
$F_{4,4,4,2}$	31.486339984158
$F_{4,4,4,3}$	9.486273186144
$F_{4,4,4,4}$	35.092661282999
$F_{5,1,1,1}$	2.436673542813
$F_{5,2,1,1}$	-0.187560043434
$F_{5,2,2,1}$	1.894897940675
$F_{5,2,2,2}$	-7.985955076347
$F_{5,3,1,1}$	-3.271570578095
$F_{5,3,2,1}$	1.096057152232
$F_{5,3,2,2}$	13.937938000022
$F_{5,3,3,1}$	-2.462590476065
$F_{5,3,3,2}$	8.141363373667
$F_{5,3,3,3}$	-10.594231926130

$F_{5,4,1,1}$	3.569021759848
$F_{5,4,2,1}$	2.701340545304
$F_{5,4,2,2}$	-9.991678016418
$F_{5,4,3,1}$	-1.457485702714
$F_{5,4,3,2}$	15.992668443638
$F_{5,4,3,3}$	4.137993602957
$F_{5,4,4,1}$	6.332025629494
$F_{5,4,4,2}$	-10.967602978270
$F_{5,4,4,3}$	17.599729340752
$F_{5,4,4,4}$	-9.575234092274
$F_{5,5,1,1}$	-0.919062029966
$F_{5,5,2,1}$	3.153215271708
$F_{5,5,2,2}$	3.614022850359
$F_{5,5,3,1}$	-4.318542503375
$F_{5,5,3,2}$	39.701644808803
$F_{5,5,3,3}$	-15.048369649589
$F_{5,5,4,1}$	5.194985560466
$F_{5,5,4,2}$	5.695654812910
$F_{5,5,4,3}$	39.613138594868
$F_{5,5,4,4}$	7.118401095172
$F_{5,5,5,1}$	9.593971117882
$F_{5,5,5,2}$	78.666289061756
$F_{5,5,5,3}$	61.379164136641
$F_{5,5,5,4}$	88.113556058182
$F_{5,5,5,5}$	379.021117134932
$F_{6,6,1,1}$	-0.021150691023
$F_{6,6,2,1}$	0.424049817081
$F_{6,6,2,2}$	-0.443840908751
$F_{6,6,3,1}$	0.046155239127
$F_{6,6,3,2}$	-0.011317929404
$F_{6,6,3,3}$	-0.254001562584
$F_{6,6,4,1}$	0.244259013415
$F_{6,6,4,2}$	0.224541646957
$F_{6,6,4,3}$	-0.174018596477
$F_{6,6,4,4}$	-0.143347172980
$F_{6,6,5,1}$	0.773240151034
$F_{6,6,5,2}$	0.045177504543
$F_{6,6,5,3}$	-0.300228695434
$F_{6,6,5,4}$	-0.039160362177
$F_{6,6,5,5}$	0.131177887351
$F_{6,6,6,6}$	0.052307194813

Table S2: C(O)OC Force Constants

$F_{1,1,0,0}$	15.023846183669
$F_{2,1,0,0}$	1.462403198572
$F_{2,2,0,0}$	3.346629139121
$F_{3,1,0,0}$	-0.096129135831
$F_{3,2,0,0}$	2.184811491477
$F_{3,3,0,0}$	5.871737144875
$F_{4,1,0,0}$	0.119807637547
$F_{4,2,0,0}$	0.570080270357
$F_{4,3,0,0}$	-0.165996595882
$F_{4,4,0,0}$	1.106201868138
$F_{5,1,0,0}$	0.040760816240
$F_{5,2,0,0}$	3.291930955282
$F_{5,3,0,0}$	2.891529489643
$F_{5,4,0,0}$	0.287809578898
$F_{5,5,0,0}$	7.385053774333
$F_{6,6,0,0}$	0.267217148896
$F_{1,1,1,0}$	-106.029782952037
$F_{2,1,1,0}$	-4.120034932080

$F_{2,2,1,0}$	-0.949206748159
$F_{2,2,2,0}$	-26.596335774714
$F_{3,1,1,0}$	0.031990003766
$F_{3,2,1,0}$	-0.608727826777
$F_{3,2,2,0}$	-2.466826308173
$F_{3,3,1,0}$	0.472535932844
$F_{3,3,2,0}$	-5.631733619106
$F_{3,3,3,0}$	-34.382667989725
$F_{4,1,1,0}$	-0.180767225879
$F_{4,2,1,0}$	-0.789273017118
$F_{4,2,2,0}$	0.770536588411
$F_{4,3,1,0}$	0.166385703960
$F_{4,3,2,0}$	-1.109387097530
$F_{4,3,3,0}$	0.554308429050
$F_{4,4,1,0}$	-1.729494571065
$F_{4,4,2,0}$	-1.896547266760
$F_{4,4,3,0}$	0.397889712455
$F_{4,4,4,0}$	-0.817322973479
$F_{5,1,1,0}$	-0.405796203951
$F_{5,2,1,0}$	-2.344507321329
$F_{5,2,2,0}$	-7.823459453568
$F_{5,3,1,0}$	1.900448665987
$F_{5,3,2,0}$	-7.362774796784
$F_{5,3,3,0}$	-4.927439788697
$F_{5,4,1,0}$	0.614989923629
$F_{5,4,2,0}$	1.499604210996
$F_{5,4,3,0}$	-1.456549080433
$F_{5,4,4,0}$	-0.914056069226
$F_{5,5,1,0}$	-1.092462954470
$F_{5,5,2,0}$	-19.857858771652
$F_{5,5,3,0}$	-15.017814832873
$F_{5,5,4,0}$	0.888054020395
$F_{5,5,5,0}$	-56.717447396684
$F_{6,6,1,0}$	-0.399257019920
$F_{6,6,2,0}$	-0.094415409977
$F_{6,6,3,0}$	-0.054445669789
$F_{6,6,4,0}$	-0.041460859393
$F_{6,6,5,0}$	-0.107956935989
$F_{1,1,1,1}$	613.507539873684
$F_{2,1,1,1}$	8.385031475487
$F_{2,2,1,1}$	5.081319986367
$F_{2,2,2,1}$	0.259931091448
$F_{2,2,2,2}$	121.419796645773
$F_{3,1,1,1}$	0.000204326340
$F_{3,2,1,1}$	2.519265703407
$F_{3,2,2,1}$	0.179217069714
$F_{3,2,2,2}$	17.224908003272
$F_{3,3,1,1}$	-2.147015749649
$F_{3,3,2,1}$	-0.880221388353
$F_{3,3,2,2}$	2.299792148062
$F_{3,3,3,1}$	2.404091840619
$F_{3,3,3,2}$	11.029878183020
$F_{3,3,3,3}$	195.743896331034
$F_{4,1,1,1}$	-0.597729253968
$F_{4,2,1,1}$	1.689252858985
$F_{4,2,2,1}$	1.817542747435
$F_{4,2,2,2}$	-2.316422701579
$F_{4,3,1,1}$	-0.422250980819
$F_{4,3,2,1}$	0.099839732279
$F_{4,3,2,2}$	-2.977553910370
$F_{4,3,3,1}$	-0.095349720954
$F_{4,3,3,2}$	4.683249928072
$F_{4,3,3,3}$	-3.962230636405
$F_{4,4,1,1}$	1.178303928683
$F_{4,4,2,1}$	3.830702590650

$F_{4,4,2,2}$	2.262109689917
$F_{4,4,3,1}$	0.258533383505
$F_{4,4,3,2}$	0.352922741329
$F_{4,4,3,3}$	-2.107565890317
$F_{4,4,4,1}$	1.104872761414
$F_{4,4,4,2}$	1.833892427494
$F_{4,4,4,3}$	0.338062081774
$F_{4,4,4,4}$	3.606489679342
$F_{5,1,1,1}$	2.437037482165
$F_{5,2,1,1}$	2.382465297311
$F_{5,2,2,1}$	6.766637670852
$F_{5,2,2,2}$	-28.363273934773
$F_{5,3,1,1}$	-3.334498916731
$F_{5,3,2,1}$	-0.227582272348
$F_{5,3,2,2}$	31.705355708811
$F_{5,3,3,1}$	-2.485403624803
$F_{5,3,3,2}$	8.703032477840
$F_{5,3,3,3}$	-10.669924070152
$F_{5,4,1,1}$	-2.635931704665
$F_{5,4,2,1}$	-2.253587674494
$F_{5,4,2,2}$	0.874461073297
$F_{5,4,3,1}$	1.825889957403
$F_{5,4,3,2}$	-1.808886685769
$F_{5,4,3,3}$	2.844192586912
$F_{5,4,4,1}$	1.428675599104
$F_{5,4,4,2}$	1.203636276260
$F_{5,4,4,3}$	-0.265319284055
$F_{5,4,4,4}$	-0.485063001588
$F_{5,5,1,1}$	-0.967975893798
$F_{5,5,2,1}$	5.813287477986
$F_{5,5,2,2}$	11.000993048872
$F_{5,5,3,1}$	-4.304092341692
$F_{5,5,3,2}$	56.046926270403
$F_{5,5,3,3}$	-15.048713016114
$F_{5,5,4,1}$	-1.492234011241
$F_{5,5,4,2}$	-1.772503773778
$F_{5,5,4,3}$	0.058297367442
$F_{5,5,4,4}$	-0.354927959975
$F_{5,5,5,1}$	9.555286284065
$F_{5,5,5,2}$	117.942518574570
$F_{5,5,5,3}$	61.284061341174
$F_{5,5,5,4}$	-6.662577745709
$F_{5,5,5,5}$	379.013771138610
$F_{6,6,1,1}$	-0.085428774122
$F_{6,6,2,1}$	0.382932773053
$F_{6,6,2,2}$	-0.015781629336
$F_{6,6,3,1}$	0.102976321184
$F_{6,6,3,2}$	-0.084322237703
$F_{6,6,3,3}$	-0.309390460471
$F_{6,6,4,1}$	0.038025639424
$F_{6,6,4,2}$	-0.148894630905
$F_{6,6,4,3}$	0.043642806670
$F_{6,6,4,4}$	-0.465715171818
$F_{6,6,5,1}$	0.770482930980
$F_{6,6,5,2}$	-0.064153775112
$F_{6,6,5,3}$	-0.260019927203
$F_{6,6,5,4}$	0.076886972207
$F_{6,6,5,5}$	0.147995761765
$F_{6,6,6,6}$	0.085369898213

Table S3: c-C_2O_2 Force Constants

$F_{1,1,0,0}$	6.144290687282
$F_{2,1,0,0}$	0.506301066671
$F_{2,2,0,0}$	4.018328021919
$F_{3,1,0,0}$	-0.880295789094
$F_{3,2,0,0}$	0.476738180662
$F_{3,3,0,0}$	0.668832744253
$F_{4,4,0,0}$	0.575469297143
$F_{5,5,0,0}$	4.566411262153
$F_{6,6,0,0}$	4.744159089462
$F_{1,1,1,0}$	-20.023836667994
$F_{2,1,1,0}$	-2.844149149854
$F_{2,2,1,0}$	-5.728169023618
$F_{2,2,2,0}$	-5.545395393502
$F_{3,1,1,0}$	2.279670225683
$F_{3,2,1,0}$	-1.147157989024
$F_{3,2,2,0}$	0.115805543964
$F_{3,3,1,0}$	0.939623456961
$F_{3,3,2,0}$	1.052360822168
$F_{3,3,3,0}$	-3.187075234020
$F_{4,4,1,0}$	-15.657562263342
$F_{4,4,2,0}$	-0.193966360119
$F_{4,4,3,0}$	1.979613942702
$F_{5,5,1,0}$	-15.016605304680
$F_{5,5,2,0}$	-2.753789129591
$F_{5,5,3,0}$	2.211362572890
$F_{6,5,4,0}$	-12.897828871186
$F_{6,6,1,0}$	-13.871425814986
$F_{6,6,2,0}$	3.094628496373
$F_{6,6,3,0}$	3.075822443602
$F_{1,1,1,1}$	55.728802323570
$F_{2,1,1,1}$	9.427934700668
$F_{2,2,1,1}$	6.506666983001
$F_{2,2,2,1}$	7.173222650401
$F_{2,2,2,2}$	7.013726576884
$F_{3,1,1,1}$	-5.345994823154
$F_{3,2,1,1}$	2.330884806010
$F_{3,2,2,1}$	1.379717591864
$F_{3,2,2,2}$	-7.154849449354
$F_{3,3,1,1}$	-3.026696370397
$F_{3,3,2,1}$	-1.896937368771
$F_{3,3,2,2}$	3.275734671131
$F_{3,3,3,1}$	3.704427611122
$F_{3,3,3,2}$	-5.899865619615
$F_{3,3,3,3}$	-3.158543255830
$F_{4,4,1,1}$	55.637043394567
$F_{4,4,2,1}$	-0.629048065487
$F_{4,4,2,2}$	-16.214687133303
$F_{4,4,3,1}$	-5.469765738116
$F_{4,4,3,2}$	3.335930005115
$F_{4,4,3,3}$	-9.012541185509
$F_{4,4,4,4}$	86.436379729573
$F_{5,5,1,1}$	44.401189833975
$F_{5,5,2,1}$	3.336202451819
$F_{5,5,2,2}$	-5.278731304746
$F_{5,5,3,1}$	-4.940048379166
$F_{5,5,3,2}$	0.289728204883
$F_{5,5,3,3}$	-6.674596811052
$F_{5,5,4,4}$	32.644834440927
$F_{5,5,5,5}$	24.954105676999
$F_{6,5,4,1}$	49.471241501957
$F_{6,5,4,2}$	3.153889196585
$F_{6,5,4,3}$	-6.831392012890
$F_{6,6,1,1}$	39.484935855710

$F_{6,6,2,1}$	2.082756026583
$F_{6,6,2,2}$	-2.512810057511
$F_{6,6,3,1}$	-7.209767367066
$F_{6,6,3,2}$	4.649541858384
$F_{6,6,3,3}$	-0.023393203145
$F_{6,6,4,4}$	52.294338925882
$F_{6,6,5,5}$	32.702117371759
$F_{6,6,6,6}$	-26.046037786948

Table S4: Si(O)OSi Force Constants

$F_{1,1,0,0}$	8.888514564084
$F_{2,1,0,0}$	0.021106719732
$F_{2,2,0,0}$	3.136944072899
$F_{3,1,0,0}$	-0.012850770740
$F_{3,2,0,0}$	1.402690323127
$F_{3,3,0,0}$	4.462801171588
$F_{4,1,0,0}$	0.145508301209
$F_{4,2,0,0}$	0.385166358749
$F_{4,3,0,0}$	-0.232450095371
$F_{4,4,0,0}$	0.656200219994
$F_{5,1,0,0}$	-0.071849575760
$F_{5,2,0,0}$	1.068542462600
$F_{5,3,0,0}$	1.756356561283
$F_{5,4,0,0}$	0.243211050779
$F_{5,5,0,0}$	3.081100969213
$F_{6,6,0,0}$	0.143227893510
$F_{1,1,1,0}$	-52.958798328488
$F_{2,1,1,0}$	-0.247619356347
$F_{2,2,1,0}$	-0.111948955891
$F_{2,2,2,0}$	-22.127064757959
$F_{3,1,1,0}$	-0.096495884735
$F_{3,2,1,0}$	0.056736702963
$F_{3,2,2,0}$	-0.709203140326
$F_{3,3,1,0}$	-0.065309157885
$F_{3,3,2,0}$	-2.665460753613
$F_{3,3,3,0}$	-24.096481051791
$F_{4,1,1,0}$	-0.198443178073
$F_{4,2,1,0}$	-0.345435711190
$F_{4,2,2,0}$	-0.178638182668
$F_{4,3,1,0}$	0.107272106342
$F_{4,3,2,0}$	-0.064042126389
$F_{4,3,3,0}$	0.148078284496
$F_{4,4,1,0}$	-0.552703963666
$F_{4,4,2,0}$	-0.642931949085
$F_{4,4,3,0}$	0.012755736649
$F_{4,4,4,0}$	-0.465935265909
$F_{5,1,1,0}$	-0.236023391821
$F_{5,2,1,0}$	-0.184175010226
$F_{5,2,2,0}$	-3.203893654837
$F_{5,3,1,0}$	-0.005403523281
$F_{5,3,2,0}$	-2.273749360995
$F_{5,3,3,0}$	-3.225756451132
$F_{5,4,1,0}$	-0.006522679160
$F_{5,4,2,0}$	0.308336948715
$F_{5,4,3,0}$	-0.184221595260
$F_{5,4,4,0}$	-0.273430060252
$F_{5,5,1,0}$	-0.147288283792
$F_{5,5,2,0}$	-6.507325029478
$F_{5,5,3,0}$	-6.204083354151
$F_{5,5,4,0}$	0.098669034551
$F_{5,5,5,0}$	-19.265993818618

$F_{6,6,1,0}$	-0.103772458379
$F_{6,6,2,0}$	-0.086549800465
$F_{6,6,3,0}$	0.023711382110
$F_{6,6,4,0}$	0.026507654303
$F_{6,6,5,0}$	-0.101249858502
$F_{1,1,1,1}$	264.601953836922
$F_{2,1,1,1}$	0.079362395183
$F_{2,2,1,1}$	1.103024856515
$F_{2,2,2,1}$	0.845021492167
$F_{2,2,2,2}$	100.638379135728
$F_{3,1,1,1}$	0.473976546064
$F_{3,2,1,1}$	-0.248137044302
$F_{3,2,2,1}$	0.053357958664
$F_{3,2,2,2}$	2.452282552170
$F_{3,3,1,1}$	-0.040561261106
$F_{3,3,2,1}$	0.101127124530
$F_{3,3,2,2}$	2.934531630770
$F_{3,3,3,1}$	0.102596240001
$F_{3,3,3,2}$	2.273719749051
$F_{3,3,3,3}$	118.340743232783
$F_{4,1,1,1}$	0.082112999623
$F_{4,2,1,1}$	0.087874742588
$F_{4,2,2,1}$	-0.164577574213
$F_{4,2,2,2}$	-1.032062336582
$F_{4,3,1,1}$	0.427026121723
$F_{4,3,2,1}$	-0.047957953128
$F_{4,3,2,2}$	1.224193822113
$F_{4,3,3,1}$	0.246472099542
$F_{4,3,3,2}$	0.313034900015
$F_{4,3,3,3}$	0.592127294660
$F_{4,4,1,1}$	-0.198602058884
$F_{4,4,2,1}$	1.025665984387
$F_{4,4,2,2}$	0.485930828684
$F_{4,4,3,1}$	0.241056009558
$F_{4,4,3,2}$	-0.044679071412
$F_{4,4,3,3}$	-0.325226157074
$F_{4,4,4,1}$	0.925869604913
$F_{4,4,4,2}$	0.756977705769
$F_{4,4,4,3}$	0.088739428000
$F_{4,4,4,4}$	1.755642429848
$F_{5,1,1,1}$	-0.073754015832
$F_{5,2,1,1}$	-0.041416507778
$F_{5,2,2,1}$	0.180417812532
$F_{5,2,2,2}$	-3.225520809532
$F_{5,3,1,1}$	-0.648970172350
$F_{5,3,2,1}$	0.424209867294
$F_{5,3,2,2}$	7.672460819687
$F_{5,3,3,1}$	1.106248460593
$F_{5,3,3,2}$	5.707013941756
$F_{5,3,3,3}$	-1.142313592839
$F_{5,4,1,1}$	-0.874559145028
$F_{5,4,2,1}$	-0.517073903227
$F_{5,4,2,2}$	-1.174772682606
$F_{5,4,3,1}$	0.727216918991
$F_{5,4,3,2}$	0.342245380799
$F_{5,4,3,3}$	-1.620830065777
$F_{5,4,4,1}$	0.621049644828
$F_{5,4,4,2}$	-0.108073215219
$F_{5,4,4,3}$	-0.503126334840
$F_{5,4,4,4}$	-0.281848571252
$F_{5,5,1,1}$	-0.292849361611
$F_{5,5,2,1}$	0.268027250608
$F_{5,5,2,2}$	4.320894753533
$F_{5,5,3,1}$	0.635699879398
$F_{5,5,3,2}$	15.337453981708

$F_{5,5,3,3}$	3.221179169317
$F_{5,5,4,1}$	-0.173361803029
$F_{5,5,4,2}$	-0.537225653417
$F_{5,5,4,3}$	-0.520635891935
$F_{5,5,4,4}$	-0.340403004419
$F_{5,5,5,1}$	1.970657382656
$F_{5,5,5,2}$	29.279126747182
$F_{5,5,5,3}$	27.327572854401
$F_{5,5,5,4}$	-2.692939254157
$F_{5,5,5,5}$	101.348402507844
$F_{6,6,1,1}$	-0.350253563832
$F_{6,6,2,1}$	-0.310737974495
$F_{6,6,2,2}$	0.343270084455
$F_{6,6,3,1}$	0.616817861349
$F_{6,6,3,2}$	-0.150953541866
$F_{6,6,3,3}$	0.194021213085
$F_{6,6,4,1}$	0.287620644211
$F_{6,6,4,2}$	-0.022844990567
$F_{6,6,4,3}$	0.152225112967
$F_{6,6,4,4}$	0.200051278870
$F_{6,6,5,1}$	-1.220337284670
$F_{6,6,5,2}$	-1.166868535941
$F_{6,6,5,3}$	-0.570039395345
$F_{6,6,5,4}$	0.912009436852
$F_{6,6,5,5}$	-0.361593245021
$F_{6,6,6,6}$	-0.075363012381

Table S5: c-Si_2O_2 Force Constants

$F_{1,1,0,0}$	4.350447425724
$F_{2,1,0,0}$	-0.198394300695
$F_{2,2,0,0}$	2.094913742715
$F_{3,3,0,0}$	1.980375422923
$F_{4,4,0,0}$	0.229997430540
$F_{5,5,0,0}$	3.867805374562
$F_{6,6,0,0}$	3.942531087669
$F_{1,1,1,0}$	-11.516337862792
$F_{2,1,1,0}$	0.022022813881
$F_{2,2,1,0}$	-2.118226963454
$F_{2,2,2,0}$	-0.353687366606
$F_{3,3,1,0}$	-11.262445549545
$F_{3,3,2,0}$	0.535829518041
$F_{4,4,1,0}$	1.380036809300
$F_{4,4,2,0}$	-0.056911778451
$F_{5,5,1,0}$	-9.387241116609
$F_{5,5,2,0}$	-0.471549024622
$F_{6,5,3,0}$	-10.033974099665
$F_{6,6,1,0}$	-9.555603417617
$F_{6,6,2,0}$	1.476471147455
$F_{1,1,1,1}$	27.416231792620
$F_{2,1,1,1}$	0.595765779548
$F_{2,2,1,1}$	2.730152728978
$F_{2,2,2,1}$	-0.162636628443
$F_{2,2,2,2}$	6.097071492116
$F_{3,3,1,1}$	31.544731709657
$F_{3,3,2,1}$	-1.267316337003
$F_{3,3,2,2}$	-4.853526668049
$F_{3,3,3,3}$	45.602872874892
$F_{4,4,1,1}$	-2.607541355869
$F_{4,4,2,1}$	1.002402650758
$F_{4,4,2,2}$	0.257414252749
$F_{4,4,3,3}$	-4.727328226602

$F_{4,4,4,4}$	3.445543503331
$F_{5,5,1,1}$	22.172039623577
$F_{5,5,2,1}$	-0.310337240139
$F_{5,5,2,2}$	-3.208508023587
$F_{5,5,3,3}$	26.024552991015
$F_{5,5,4,4}$	-3.708162334288
$F_{5,5,5,5}$	7.688744310625
$F_{6,5,3,1}$	27.912398969239
$F_{6,5,3,2}$	-0.749887948318
$F_{6,5,4,3}$	0.325242098515
$F_{6,6,1,1}$	22.627498598712
$F_{6,6,2,1}$	-0.133764787208
$F_{6,6,2,2}$	-2.043271386450
$F_{6,6,3,3}$	28.279171170424
$F_{6,6,4,4}$	-3.892859893945
$F_{6,6,5,5}$	19.898142848520
$F_{6,6,6,6}$	9.724222396484

Table S6: C(O)OC Frequencies and ZPVE with motion coupling

	Description	Frequencies [cm^{-1}]
ω_1	O_1-C_1 stretch	2030.4
ω_2	O_2-C_2 stretch	1236.1
ω_3	$C_1-O_2-C_2$ bend	947.4
ω_4	(C_1-O_2 stretch) + ($O_1-C_1-O_2$ bend)	518.1
ω_5	out-of-plane bend	508.2
ω_6	(C_1-O_2 stretch) - ($O_1-C_1-O_2$ bend)	478.3
ν_1	O_1-C_1 stretch	2006.9
ν_2	O_2-C_2 stretch	1248.9
ν_3	$C_1-O_2-C_2$ bend	919.6
ν_4	(C_1-O_2 stretch) + ($O_1-C_1-O_2$ bend)	454.2
ν_5	out-of-plane bend	492.4
ν_6	(C_1-O_2 stretch) - ($O_1-C_1-O_2$ bend)	310.6
ZPVE [cm^{-1}]		2808.9

Table S7: C(O)OC Rotational Constants [MHz] with motion coupling

A_e	38841.1
B_e	8384.9
C_e	6896.1
A_0	38826.2
B_0	8274.5
C_0	6807.3
κ	-0.90835
A_1	38818.2
B_1	8232.2
C_1	6778.5
A_2	38374.6
B_2	8298.3
C_2	6811.8
A_3	38967.6
B_3	8252.8
C_3	6792.9
A_4	40153.9
B_4	8217.8
C_4	6755.1
A_5	42481.4
B_5	8278.3
C_5	6818.7
A_6	34131.2
B_6	8147.1
C_6	6709.0

Table S8: C(O)OC Quartic and Sextic Distortion Constants with motion coupling

Constant	Units	
Δ_J	kHz	3.456
Δ_K	kHz	151.182
Δ_{JK}	kHz	51.193
δ_J	Hz	682.262
δ_K	kHz	36.183
Φ_J	mHz	-11.976
Φ_K	Hz	-2.507
Φ_{JK}	mHz	-344.913
Φ_{KJ}	Hz	2.500
ϕ_j	mHz	-3.170
ϕ_{jk}	mHz	-257.387
ϕ_k	Hz	3.710

Table S9: C(O)OC Geometry [Å or Deg] with motion coupling

Coordinate	r_e	r_0
$r(O_1-C_2)$	1.1717212	1.1703507
$r(C_2-O_3)$	1.4387036	1.4719814
$r(O_3-C_4)$	1.3594921	1.3566819
$\angle(C_2-O_1-O_3)$	134.4045544	133.3263362
$\angle(O_3-C_2-C_4)$	62.0919164	61.6193430

Table S10: C(O)OC Fermi Resonances with motion coupling

$2\nu_3 = \nu_3 + \nu_2 = \nu_1$
$\nu_6 + \nu_3 = \nu_2$
$2\nu_4 = 2\nu_5 = 2\nu_6 = \nu_6 + \nu_4 = \nu_3$

Table S11: C(O)OC Geometry [Å or Deg]

Coordinate	r_e	r_0
$r(O_1{-}C_2)$	1.1717212	1.1703483
$r(C_2{-}O_3)$	1.4387035	1.4720071
$r(O_3{-}C_4)$	1.3594921	1.3566750
$\angle(C_2{-}O_1{-}O_3)$	134.4045563	133.3254266
$\angle(O_3{-}C_2{-}C_4)$	62.0919179	61.6188493

Table S12: C(O)OC Fermi Resonances

$2\nu_3 = \nu_3 + \nu_2 = \nu_1$
$\nu_6 + \nu_3 = \nu_2$
$2\nu_4 = 2\nu_5 = 2\nu_6 = \nu_6 + \nu_4 = \nu_3$

Table S13: $^{12}C(^{16}O)^{16}O^{13}C$ Frequencies and ZPVE

	Description	Frequencies [cm^{-1}]
ω_1	$O_1{-}C_1$ stretch	2027.5
ω_2	$O_2{-}C_2$ stretch	1210.9
ω_3	$C_1{-}O_2{-}C_2$ bend	933.2
ω_4	($C_1{-}O_2$ stretch) + ($O_1{-}C_1{-}O_2$ bend)	514.8
ω_5	out-of-plane bend	506.2
ω_6	($C_1{-}O_2$ stretch) - ($O_1{-}C_1{-}O_2$ bend)	474.4
ν_1	$O_1{-}C_1$ stretch	2004.0
ν_2	$O_2{-}C_2$ stretch	1225.7
ν_3	$C_1{-}O_2{-}C_2$ bend	906.4
ν_4	($C_1{-}O_2$ stretch) + ($O_1{-}C_1{-}O_2$ bend)	445.9
ν_5	out-of-plane bend	490.9
ν_6	($C_1{-}O_2$ stretch) - ($O_1{-}C_1{-}O_2$ bend)	313.7
ZPVE [cm^{-1}]		2782.8

Table S14: $^{12}C(^{16}O)^{16}O^{13}C$ Rotational Constants [MHz]

A_e	37443.5
B_e	8235.0
C_e	6750.4
A_0	37442.9
B_0	8128.7
C_0	6665.5
κ	-0.90492
A_1	37439.2
B_1	8086.0
C_1	6636.8
A_2	37008.8
B_2	8150.8
C_2	6668.6
A_3	37596.4
B_3	8105.5
C_3	6651.3
A_4	38546.0
B_4	8070.3
C_4	6614.0
A_5	40767.8
B_5	8132.8
C_5	6676.7
A_6	33298.6
B_6	8013.9
C_6	6575.6

Table S15: $^{12}C(^{16}O)^{16}O^{13}C$ Quartic and Sextic Distortion Constants

Constant	Units	
Δ_J	kHz	3.220
Δ_K	kHz	142.886
Δ_{JK}	kHz	49.644
δ_J	Hz	645.073
δ_K	kHz	34.593
Φ_J	mHz	-10.040
Φ_K	Hz	-2.308
Φ_{JK}	mHz	-328.667
Φ_{KJ}	Hz	2.355
ϕ_j	mHz	-2.688
ϕ_{jk}	mHz	-235.391
ϕ_k	Hz	3.452

Table S16: $^{12}C(^{16}O)^{16}O^{13}C$ Geometry [Å or Deg]

Coordinate	r_e	r_0
$r(O_1-C_2)$	1.1717212	1.1703046
$r(C_2-O_3)$	1.4387035	1.4720723
$r(O_3-C_4)$	1.3594921	1.3565218
$\angle(C_2-O_1-O_3)$	134.4045563	133.3281560
$\angle(O_3-C_2-C_4)$	62.0919179	61.6165354

Table S17: $^{12}C(^{16}O)^{16}O^{13}C$ Fermi Resonances

$2\nu_3 = \nu_3 + \nu_2 = \nu_1$
$2\nu_5 = \nu_6 + \nu_3 = \nu_2$
$2\nu_4 = 2\nu_5 = 2\nu_6 = \nu_6 + \nu_4 = \nu_3$

Table S18: $^{12}C(^{16}O)^{18}O^{12}C$ Frequencies and ZPVE

	Description	Frequencies [cm^{-1}]
ω_1	O_1-C_1 stretch	2030.4
ω_2	O_2-C_2 stretch	1209.8
ω_3	$C_1-O_2-C_2$ bend	939.4
ω_4	(C_1-O_2 stretch) + ($O_1-C_1-O_2$ bend)	508.0
ω_5	out-of-plane bend	501.9
ω_6	(C_1-O_2 stretch) - ($O_1-C_1-O_2$ bend)	475.6
ν_1	O_1-C_1 stretch	2006.1
ν_2	O_2-C_2 stretch	1208.6
ν_3	$C_1-O_2-C_2$ bend	910.8
ν_4	(C_1-O_2 stretch) + ($O_1-C_1-O_2$ bend)	493.0
ν_5	out-of-plane bend	468.7
ν_6	(C_1-O_2 stretch) - ($O_1-C_1-O_2$ bend)	285.7
ZPVE [cm^{-1}]		2783.6

Table S19: $^{12}C(^{16}O)^{18}O^{12}C$ Rotational Constants [MHz]

A_e	36682.3
B_e	8176.8
C_e	6686.3
A_0	36681.0
B_0	8067.0
C_0	6599.6
κ	-0.90244
A_1	36665.2
B_1	8027.7
C_1	6573.0
A_2	36277.6
B_2	8088.7
C_2	6603.6
A_3	36792.9
B_3	8049.0
C_3	6586.6
A_4	39952.8
B_4	8084.7
C_4	6610.3
A_5	36895.2
B_5	8026.0
C_5	6573.3
A_6	33499.7
B_6	7906.2
C_6	6477.7

Table S20: $^{12}C(^{16}O)^{18}O^{12}C$ Quartic and Sextic Distortion Constants

Constant	Units	
Δ_J	kHz	3.468
Δ_K	kHz	131.734
Δ_{JK}	kHz	46.461
δ_J	Hz	705.886
δ_K	kHz	33.896
Φ_J	mHz	-13.394
Φ_K	Hz	-2.343
Φ_{JK}	mHz	-295.573
Φ_{KJ}	Hz	2.281
ϕ_j	mHz	-3.732
ϕ_{jk}	mHz	-246.490
ϕ_k	Hz	3.228

Table S21: $^{12}C(^{16}O)^{18}O^{12}C$ Geometry [Å or Deg]

Coordinate	r_e	r_0
$r(O_1-C_2)$	1.1717212	1.1704133
$r(C_2-O_3)$	1.4387035	1.4712782
$r(O_3-C_4)$	1.3594921	1.3567044
$\angle(C_2-O_1-O_3)$	134.4045563	133.3380614
$\angle(O_3-C_2-C_4)$	62.0919179	61.6352607

Table S22: $^{12}C(^{16}O)^{18}O^{12}C$ Fermi Resonances

$2\nu_3 = \nu_3 + \nu_2 = \nu_1$
$2\nu_4 = \nu_2$
$2\nu_4 = 2\nu_5 = 2\nu_6 = \nu_6 + \nu_5 = \nu_3$

Table S23: $^{12}C(^{18}O)^{16}O^{12}C$ Frequencies and ZPVE

	Description	Frequencies [cm^{-1}]
ω_1	O_1-C_1 stretch	1996.5
ω_2	O_2-C_2 stretch	1231.7
ω_3	$C_1-O_2-C_2$ bend	931.3
ω_4	(C_1-O_2 stretch) + ($O_1-C_1-O_2$ bend)	512.2
ω_5	out-of-plane bend	503.7
ω_6	(C_1-O_2 stretch) - ($O_1-C_1-O_2$ bend)	475.0
ν_1	O_1-C_1 stretch	1973.6
ν_2	O_2-C_2 stretch	1242.0
ν_3	$C_1-O_2-C_2$ bend	904.0
ν_4	(C_1-O_2 stretch) + ($O_1-C_1-O_2$ bend)	439.5
ν_5	out-of-plane bend	488.7
ν_6	(C_1-O_2 stretch) - ($O_1-C_1-O_2$ bend)	318.2
ZPVE [cm^{-1}]		2775.4

Table S24: $^{12}C(^{18}O)^{16}O^{12}C$ Rotational Constants [MHz]

A_e	38835.7
B_e	7856.1
C_e	6534.3
A_0	38815.3
B_0	7755.2
C_0	6451.5
κ	-0.91943
A_1	38810.6
B_1	7714.0
C_1	6423.0
A_2	38357.0
B_2	7778.4
C_2	6457.0
A_3	38966.3
B_3	7733.7
C_3	6436.8
A_4	39839.2
B_4	7694.4
C_4	6396.8
A_5	43117.7
B_5	7759.7
C_5	6462.4
A_6	33760.5
B_6	7649.1
C_6	6367.2

Table S25: $^{12}C(^{18}O)^{16}O^{12}C$ Quartic and Sextic Distortion Constants

Constant	Units	
Δ_J	kHz	3.000
Δ_K	kHz	156.337
Δ_{JK}	kHz	46.223
δ_J	Hz	562.919
δ_K	kHz	32.658
Φ_J	mHz	-9.191
Φ_K	Hz	-2.177
Φ_{JK}	mHz	-274.488
Φ_{KJ}	Hz	2.115
ϕ_j	mHz	-2.295
ϕ_{jk}	mHz	-204.119
ϕ_k	Hz	3.464

Table S26: $^{12}C(^{18}O)^{16}O^{12}C$ Geometry [Å or Deg]

Coordinate	r_e	r_0
$r(O_1-C_2)$	1.1717212	1.1703408
$r(C_2-O_3)$	1.4387035	1.4719488
$r(O_3-C_4)$	1.3594921	1.3566623
$\angle(C_2-O_1-O_3)$	134.4045563	133.3236999
$\angle(O_3-C_2-C_4)$	62.0919179	61.6165057

Table S27: $^{12}C(^{18}O)^{16}O^{12}C$ Fermi Resonances

$2\nu_3 = \nu_3 + \nu_2 = \nu_1$
$\nu_6 + \nu_3 = \nu_2$
$2\nu_5 = 2\nu_6 = \nu_6 + \nu_4 = \nu_3$

Table S28: $^{12}C(^{18}O)^{18}O^{12}C$ Frequencies and ZPVE

	Description	Frequencies [cm^{-1}]
ω_1	O_1-C_1 stretch	1996.5
ω_2	O_2-C_2 stretch	1204.7
ω_3	$C_1-O_2-C_2$ bend	924.0
ω_4	(C_1-O_2 stretch) + ($O_1-C_1-O_2$ bend)	503.6
ω_5	out-of-plane bend	495.0
ω_6	(C_1-O_2 stretch) - ($O_1-C_1-O_2$ bend)	473.1
ν_1	O_1-C_1 stretch	1972.7
ν_2	O_2-C_2 stretch	1212.7
ν_3	$C_1-O_2-C_2$ bend	895.4
ν_4	(C_1-O_2 stretch) + ($O_1-C_1-O_2$ bend)	488.7
ν_5	out-of-plane bend	455.9
ν_6	(C_1-O_2 stretch) - ($O_1-C_1-O_2$ bend)	291.4
ZPVE [cm^{-1}]		2749.6

Table S29: $^{12}C(^{18}O)^{18}O^{12}C$ Rotational Constants [MHz]

A_e	36673.3
B_e	7652.8
C_e	6331.6
A_0	36666.0
B_0	7552.7
C_0	6250.9
κ	-0.91440
A_1	36653.4
B_1	7514.4
C_1	6224.4
A_2	36254.4
B_2	7574.2
C_2	6255.9
A_3	36789.0
B_3	7534.3
C_3	6237.4
A_4	40350.9
B_4	7562.1
C_4	6261.1
A_5	36879.5
B_5	7516.0
C_5	6222.2
A_6	33053.8
B_6	7414.9
C_6	6143.0

Table S30: $^{12}C(^{18}O)^{18}O^{12}C$ Quartic and Sextic Distortion Constants

Constant	Units	
Δ_J	kHz	3.002
Δ_K	kHz	136.321
Δ_{JK}	kHz	41.991
δ_J	Hz	580.245
δ_K	kHz	30.579
Φ_J	mHz	-10.228
Φ_K	Hz	-2.035
Φ_{JK}	mHz	-235.034
Φ_{KJ}	Hz	1.925
ϕ_j	mHz	-2.694
ϕ_{jk}	mHz	-195.277
ϕ_k	Hz	3.014

Table S31: $^{12}C(^{18}O)^{18}O^{12}C$ Geometry [Å or Deg]

Coordinate	r_e	r_0
$r(O_1-C_2)$	1.1717212	1.1704062
$r(C_2-O_3)$	1.4387035	1.4712159
$r(O_3-C_4)$	1.3594921	1.3566927
$\angle(C_2-O_1-O_3)$	134.4045563	133.3363692
$\angle(O_3-C_2-C_4)$	62.0919179	61.6330167

Table S32: $^{12}C(^{18}O)^{18}O^{12}C$ Fermi Resonances

$2\nu_3 = \nu_3 + \nu_2 = \nu_1$
$2\nu_4 = \nu_6 + \nu_3 = \nu_2$
$2\nu_4 = 2\nu_5 = 2\nu_6 = \nu_6 + \nu_5 = \nu_3$

Table S33: $^{13}C(^{16}O)^{16}O^{12}C$ Frequencies and ZPVE

	Description	Frequencies [cm^{-1}]
ω_1	O_1-C_1 stretch	1978.2
ω_2	O_2-C_2 stretch	1234.9
ω_3	$C_1-O_2-C_2$ bend	940.4
ω_4	(C_1-O_2 stretch) + ($O_1-C_1-O_2$ bend)	517.0
ω_5	out-of-plane bend	493.5
ω_6	(C_1-O_2 stretch) - ($O_1-C_1-O_2$ bend)	466.4
ν_1	O_1-C_1 stretch	1957.0
ν_2	O_2-C_2 stretch	1246.1
ν_3	$C_1-O_2-C_2$ bend	912.0
ν_4	(C_1-O_2 stretch) + ($O_1-C_1-O_2$ bend)	451.8
ν_5	out-of-plane bend	479.3
ν_6	(C_1-O_2 stretch) - ($O_1-C_1-O_2$ bend)	308.5
ZPVE [cm^{-1}]		2767.4

Table S34: $^{13}C(^{16}O)^{16}O^{12}C$ Rotational Constants [MHz]

A_e	38741.3
B_e	8372.9
C_e	6884.7
A_0	38706.2
B_0	8264.5
C_0	6796.8
κ	-0.90801
A_1	38698.2
B_1	8224.9
C_1	6769.9
A_2	38262.6
B_2	8288.8
C_2	6801.9
A_3	38832.3
B_3	8244.4
C_3	6783.3
A_4	39366.0
B_4	8204.4
C_4	6739.9
A_5	43600.0
B_5	8267.8
C_5	6807.8
A_6	33408.4
B_6	8140.5
C_6	6702.2

Table S35: $^{13}C(^{16}O)^{16}O^{12}C$ Quartic and Sextic Distortion Constants

Constant	Units	
Δ_J	kHz	3.479
Δ_K	kHz	147.731
Δ_{JK}	kHz	51.114
δ_J	Hz	685.213
δ_K	kHz	36.072
Φ_J	mHz	-12.356
Φ_K	Hz	-2.386
Φ_{JK}	mHz	-364.548
Φ_{KJ}	Hz	2.451
ϕ_j	mHz	-3.226
ϕ_{jk}	mHz	-265.785
ϕ_k	Hz	3.612

Table S36: $^{13}C(^{16}O)^{16}O^{12}C$ Geometry [Å or Deg]

Coordinate	r_e	r_0
$r(O_1-C_2)$	1.1717212	1.1704055
$r(C_2-O_3)$	1.4387035	1.4711731
$r(O_3-C_4)$	1.3594921	1.3569285
$\angle(C_2-O_1-O_3)$	134.4045563	133.3576373
$\angle(O_3-C_2-C_4)$	62.0919179	61.6300776

Table S37: $^{13}C(^{16}O)^{16}O^{12}C$ Fermi Resonances

$2\nu_3 = \nu_3 + \nu_2 = \nu_1$
$\nu_6 + \nu_3 = \nu_2$
$2\nu_4 = 2\nu_5 = 2\nu_6 = \nu_6 + \nu_4 = \nu_3$

Table S38: $^{13}C(^{16}O)^{16}O^{13}C$ Frequencies and ZPVE

	Description	Frequencies [cm^{-1}]
ω_1	O_1-C_1 stretch	1975.2
ω_2	O_2-C_2 stretch	1209.5
ω_3	$C_1-O_2-C_2$ bend	925.9
ω_4	(C_1-O_2 stretch) + ($O_1-C_1-O_2$ bend)	513.8
ω_5	out-of-plane bend	491.4
ω_6	(C_1-O_2 stretch) - ($O_1-C_1-O_2$ bend)	462.7
ν_1	O_1-C_1 stretch	1953.8
ν_2	O_2-C_2 stretch	1223.4
ν_3	$C_1-O_2-C_2$ bend	899.0
ν_4	(C_1-O_2 stretch) + ($O_1-C_1-O_2$ bend)	445.1
ν_5	out-of-plane bend	477.2
ν_6	(C_1-O_2 stretch) - ($O_1-C_1-O_2$ bend)	310.2
ZPVE [cm^{-1}]		2741.7

Table S39: $^{13}C(^{16}O)^{16}O^{13}C$ Rotational Constants [MHz]

A_e	37358.0
B_e	8222.1
C_e	6739.0
A_0	37338.8
B_0	8117.8
C_0	6654.9
κ	-0.90464
A_1	37334.7
B_1	8077.8
C_1	6628.0
A_2	36911.2
B_2	8140.4
C_2	6658.5
A_3	37477.2
B_3	8096.1
C_3	6641.4
A_4	37891.7
B_4	8057.8
C_4	6599.4
A_5	41707.7
B_5	8121.3
C_5	6665.7
A_6	32671.8
B_6	8004.9
C_6	6568.0

Table S40: $^{13}C(^{16}O)^{16}O^{13}C$ Quartic and Sextic Distortion Constants

Constant	Units	
Δ_J	kHz	3.241
Δ_K	kHz	139.535
Δ_{JK}	kHz	49.548
δ_J	Hz	647.794
δ_K	kHz	34.492
Φ_J	mHz	-10.369
Φ_K	Hz	-2.224
Φ_{JK}	mHz	-345.694
Φ_{KJ}	Hz	2.313
ϕ_j	mHz	-2.739
ϕ_{jk}	mHz	-242.919
ϕ_k	Hz	3.365

Table S41: $^{13}C(^{16}O)^{16}O^{13}C$ Geometry [Å or Deg]

Coordinate	r_e	r_0
$r(O_1-C_2)$	1.1717212	1.1703610
$r(C_2-O_3)$	1.4387035	1.4712391
$r(O_3-C_4)$	1.3594921	1.3567757
$\angle(C_2-O_1-O_3)$	134.4045563	133.3604061
$\angle(O_3-C_2-C_4)$	62.0919179	61.6277284

Table S42: $^{13}C(^{16}O)^{16}O^{13}C$ Fermi Resonances

$2\nu_3 = \nu_3 + \nu_2 = \nu_1$
$2\nu_4 = \nu_6 + \nu_3 = \nu_2$
$2\nu_4 = 2\nu_5 = 2\nu_6 = \nu_6 + \nu_4 = \nu_3$

Table S43: c-C_2O_2 Geometry [Å or Deg]

Coordinate	r_e	r_0
$r(O_1-C_2)$	1.3813041	1.3891670
$r(O_1-C_3)$	1.3813041	1.3891670
$r(O_1-O_4)$	1.9840284	1.9955788
$\angle(O_1-O_4-C_2)$	44.0962196	44.0885741
$\angle(O_1-O_4-C_3)$	44.0962197	44.0885743

Table S44: c-C_2O_2 Fermi Resonances

$2\nu_5 = \nu_4$

Table S45: c-$^{12}C^{12}C^{16}O^{18}O$ Frequencies and ZPVE

	Description	Frequencies [cm^{-1}]
ω_1	(O_1-O_2 stretch) + (C_1-C_2 stretch)	1228.6
ω_2	(O_1-C_1 stretch) + (O_1-C_2 stretch) - (C_1-O_2 stretch) - (C_2-O_2 stretch)	1079.8
ω_3	(O_1-C_1 stretch) - (O_1-C_2 stretch) + (C_1-O_2 stretch) - (C_2-O_2 stretch)	1042.7
ω_4	(O_1-O_2 stretch) - (C_1-C_2 stretch)	993.7
ω_5	out-of-plane bend	439.4
ω_6	(O_1-C_1 stretch) - (O_1-C_2 stretch) - (C_1-O_2 stretch) + (C_2-O_2 stretch)	370.0
ν_1	(O_1-O_2 stretch) + (C_1-C_2 stretch)	1236.6
ν_2	(O_1-C_1 stretch) + (O_1-C_2 stretch) - (C_1-O_2 stretch) - (C_2-O_2 stretch)	1016.9
ν_3	(O_1-C_1 stretch) - (O_1-C_2 stretch) + (C_1-O_2 stretch) - (C_2-O_2 stretch)	998.3
ν_4	(O_1-O_2 stretch) - (C_1-C_2 stretch)	974.9
ν_5	out-of-plane bend	366.0
ν_6	(O_1-C_1 stretch) - (O_1-C_2 stretch) - (C_1-O_2 stretch) + (C_2-O_2 stretch)	311.0
ZPVE [cm^{-1}]		2538.3

Table S46: c-$^{12}C^{12}C^{16}O^{18}O$ Rotational Constants [MHz]

A_e	23278.3
B_e	14846.6
C_e	9282.8
A_0	23049.6
B_0	14768.8
C_0	9158.0
κ	-0.19220
A_1	22996.9
B_1	14735.6
C_1	9133.4
A_2	22943.0
B_2	14755.1
C_2	9121.4
A_3	23098.8
B_3	14716.4
C_3	9136.0
A_4	23077.4
B_4	14735.5
C_4	9145.2
A_5	22827.0
B_5	14807.0
C_5	9135.3
A_6	22897.7
B_6	14707.5
C_6	9026.9

Table S47: c-$^{12}C^{12}C^{16}O^{18}O$ Quartic and Sextic Distortion Constants

Constant	Units	
Δ_J	kHz	8.995
Δ_K	kHz	-139.741
Δ_{JK}	kHz	176.181
δ_J	kHz	2.972
δ_K	kHz	100.084
Φ_J	mHz	12.872
Φ_K	Hz	6.801
Φ_{JK}	Hz	-1.047
Φ_{KJ}	Hz	-5.605
ϕ_j	mHz	6.163
ϕ_{jk}	mHz	-507.164
ϕ_k	Hz	1.397

Table S48: c-$^{12}C^{12}C^{16}O^{18}O$ Geometry [Å or Deg]

Coordinate	r_e	r_0
$r(O_1{-}C_2)$	1.3813041	1.3891216
$r(O_1{-}C_3)$	1.3813041	1.3891216
$r(O_1{-}O_4)$	1.9840284	1.9954322
$\angle(O_1{-}O_4{-}C_2)$	44.0962196	44.0860097
$\angle(O_1{-}O_4{-}C_3)$	44.0962197	44.0860099

Table S49: c-$^{12}C^{12}C^{16}O^{18}O$ Fermi Resonances

$\nu_6 + \nu_3 = \nu_1$
$2\nu_5 = \nu_4$

Table S50: c-$^{12}C^{12}C^{18}O^{18}O$ Frequencies and ZPVE

	Description	Frequencies [cm^{-1}]
ω_1	(O_1–O_2 stretch) + (C_1–C_2 stretch)	1219.9
ω_2	(O_1–C_1 stretch) + (O_1–C_2 stretch) - (C_1–O_2 stretch) - (C_2–O_2 stretch)	1063.7
ω_3	(O_1–C_1 stretch) - (O_1–C_2 stretch) + (C_1–O_2 stretch) - (C_2–O_2 stretch)	1030.2
ω_4	(O_1–O_2 stretch) - (C_1–C_2 stretch)	972.7
ω_5	out-of-plane bend	434.5
ω_6	(O_1–C_1 stretch) - (O_1–C_2 stretch) - (C_1–O_2 stretch) + (C_2–O_2 stretch)	365.9
ν_1	(O_1–O_2 stretch) + (C_1–C_2 stretch)	1228.4
ν_2	(O_1–C_1 stretch) + (O_1–C_2 stretch) - (C_1–O_2 stretch) - (C_2–O_2 stretch)	1000.8
ν_3	(O_1–C_1 stretch) - (O_1–C_2 stretch) + (C_1–O_2 stretch) - (C_2–O_2 stretch)	987.0
ν_4	(O_1–O_2 stretch) - (C_1–C_2 stretch)	957.6
ν_5	out-of-plane bend	362.8
ν_6	(O_1–C_1 stretch) - (O_1–C_2 stretch) - (C_1–O_2 stretch) + (C_2–O_2 stretch)	308.3
ZPVE [cm^{-1}]		2505.8

Table S51: c-$^{12}C^{12}C^{18}O^{18}O$ Rotational Constants [MHz]

A_e	23260.6
B_e	14000.9
C_e	8947.6
A_0	23037.5
B_0	13926.5
C_0	8829.6
κ	-0.28253
A_1	22990.0
B_1	13896.4
C_1	8807.1
A_2	22924.9
B_2	13913.9
C_2	8793.8
A_3	23086.7
B_3	13877.9
C_3	8802.1
A_4	23070.9
B_4	13893.4
C_4	8824.1
A_5	22817.9
B_5	13961.3
C_5	8809.2
A_6	22888.7
B_6	13867.4
C_6	8705.6

Table S52: c-$^{12}C^{12}C^{18}O^{18}O$ Quartic and Sextic Distortion Constants

Constant	Units	
Δ_J	kHz	8.011
Δ_K	kHz	-127.614
Δ_{JK}	kHz	164.701
δ_J	kHz	2.594
δ_K	kHz	93.688
Φ_J	mHz	10.458
Φ_K	Hz	6.301
Φ_{JK}	mHz	-955.975
Φ_{KJ}	Hz	-5.195
ϕ_j	mHz	4.988
ϕ_{jk}	mHz	-463.898
ϕ_k	Hz	1.607

Table S53: c-$^{12}C^{12}C^{18}O^{18}O$ Geometry [Å or Deg]

Coordinate	r_e	r_0
$r(O_1{-}C_2)$	1.3813041	1.3889568
$r(O_1{-}C_3)$	1.3813041	1.3889568
$r(O_1{-}O_4)$	1.9840284	1.9952869
$\angle(O_1{-}O_4{-}C_2)$	44.0962196	44.0882770
$\angle(O_1{-}O_4{-}C_3)$	44.0962197	44.0882772

Table S54: c-$^{12}C^{12}C^{18}O^{18}O$ Fermi Resonances

$2\nu_5 = \nu_4$

Table S55: c-$^{13}C^{12}C^{16}O^{16}O$ Frequencies and ZPVE

	Description	Frequencies [cm^{-1}]
ω_1	($O_1{-}O_2$ stretch) + ($C_1{-}C_2$ stretch)	1221.4
ω_2	($O_1{-}C_1$ stretch) + ($O_1{-}C_2$ stretch) - ($C_1{-}O_2$ stretch) - ($C_2{-}O_2$ stretch)	1078.9
ω_3	($O_1{-}C_1$ stretch) - ($O_1{-}C_2$ stretch) + ($C_1{-}O_2$ stretch) - ($C_2{-}O_2$ stretch)	1044.1
ω_4	($O_1{-}O_2$ stretch) - ($C_1{-}C_2$ stretch)	1012.0
ω_5	out-of-plane bend	439.0
ω_6	($O_1{-}C_1$ stretch) - ($O_1{-}C_2$ stretch) - ($C_1{-}O_2$ stretch) + ($C_2{-}O_2$ stretch)	369.9
ν_1	($O_1{-}O_2$ stretch) + ($C_1{-}C_2$ stretch)	1231.9
ν_2	($O_1{-}C_1$ stretch) + ($O_1{-}C_2$ stretch) - ($C_1{-}O_2$ stretch) - ($C_2{-}O_2$ stretch)	1014.4
ν_3	($O_1{-}C_1$ stretch) - ($O_1{-}C_2$ stretch) + ($C_1{-}O_2$ stretch) - ($C_2{-}O_2$ stretch)	1002.1
ν_4	($O_1{-}O_2$ stretch) - ($C_1{-}C_2$ stretch)	989.4
ν_5	out-of-plane bend	365.7
ν_6	($O_1{-}C_1$ stretch) - ($O_1{-}C_2$ stretch) - ($C_1{-}O_2$ stretch) + ($C_2{-}O_2$ stretch)	310.8
ZPVE [cm^{-1}]		2543.8

Table S56: c-$^{13}C^{12}C^{16}O^{16}O$ Rotational Constants [MHz]

A_e	22387.0
B_e	15727.4
C_e	9463.5
A_0	22163.0
B_0	15648.2
C_0	9334.5
κ	-0.01568
A_1	22108.3
B_1	15608.6
C_1	9307.6
A_2	22052.8
B_2	15687.1
C_2	9294.6
A_3	22214.5
B_3	15587.8
C_3	9306.6
A_4	22191.7
B_4	15570.2
C_4	9331.2
A_5	21948.7
B_5	15690.2
C_5	9309.2
A_6	22014.4
B_6	15587.2
C_6	9199.5

Table S57: c-$^{13}C^{12}C^{16}O^{16}O$ Quartic and Sextic Distortion Constants

Constant	Units	
Δ_J	kHz	10.048
Δ_K	kHz	-148.516
Δ_{JK}	kHz	180.953
δ_J	kHz	3.424
δ_K	kHz	102.581
Φ_J	mHz	16.045
Φ_K	Hz	6.833
Φ_{JK}	Hz	-1.086
Φ_{KJ}	Hz	-5.618
ϕ_j	mHz	7.725
ϕ_{jk}	mHz	-524.101
ϕ_k	mHz	851.357

Table S58: c-$^{13}C^{12}C^{16}O^{16}O$ Geometry [Å or Deg]

Coordinate	r_e	r_0
$r(O_1-C_2)$	1.3813041	1.3890322
$r(O_1-C_3)$	1.3813041	1.3891362
$r(O_1-O_4)$	1.9840284	1.9954515
$\angle(O_1-O_4-C_2)$	44.0962196	44.0865974
$\angle(O_1-O_4-C_3)$	44.0962197	44.0910515

Table S59: c-$^{13}C^{12}C^{16}O^{16}O$ Fermi Resonances

$2\nu_5 = \nu_4$

Table S60: c-$^{13}C^{13}C^{16}O^{16}O$ Frequencies and ZPVE

	Description	Frequencies [cm^{-1}]
ω_1	(O_1-O_2 stretch) + (C_1-C_2 stretch)	1204.2
ω_2	(O_1-C_1 stretch) + (O_1-C_2 stretch) - (C_1-O_2 stretch) - (C_2-O_2 stretch)	1066.9
ω_3	(O_1-C_1 stretch) - (O_1-C_2 stretch) + (C_1-O_2 stretch) - (C_2-O_2 stretch)	1030.9
ω_4	(O_1-O_2 stretch) - (C_1-C_2 stretch)	1008.0
ω_5	out-of-plane bend	433.6
ω_6	(O_1-C_1 stretch) - (O_1-C_2 stretch) - (C_1-O_2 stretch) + (C_2-O_2 stretch)	365.6
ν_1	(O_1-O_2 stretch) + (C_1-C_2 stretch)	1215.8
ν_2	(O_1-C_1 stretch) + (O_1-C_2 stretch) - (C_1-O_2 stretch) - (C_2-O_2 stretch)	1003.9
ν_3	(O_1-C_1 stretch) - (O_1-C_2 stretch) + (C_1-O_2 stretch) - (C_2-O_2 stretch)	987.3
ν_4	(O_1-O_2 stretch) - (C_1-C_2 stretch)	986.8
ν_5	out-of-plane bend	362.1
ν_6	(O_1-C_1 stretch) - (O_1-C_2 stretch) - (C_1-O_2 stretch) + (C_2-O_2 stretch)	307.8
ZPVE [cm^{-1}]		2516.6

Table S61: c-$^{13}C^{13}C^{16}O^{16}O$ Rotational Constants [MHz]

A_e	21519.4
B_e	15719.9
C_e	9307.4
A_0	21304.6
B_0	15643.2
C_0	9181.3
κ	0.06602
A_1	21249.6
B_1	15601.9
C_1	9153.6
A_2	21199.4
B_2	15636.3
C_2	9143.0
A_3	21364.5
B_3	15587.4
C_3	9153.0
A_4	21321.6
B_4	15610.1
C_4	9180.5
A_5	21100.9
B_5	15685.2
C_5	9145.2
A_6	21162.4
B_6	15584.3
C_6	9060.3

Table S62: c-$^{13}C^{13}C^{16}O^{16}O$ Quartic and Sextic Distortion Constants

Constant	Units	
Δ_J	kHz	10.031
Δ_K	kHz	-144.671
Δ_{JK}	kHz	174.031
δ_J	kHz	3.458
δ_K	kHz	98.625
Φ_J	mHz	16.361
Φ_K	Hz	6.365
Φ_{JK}	Hz	-1.030
Φ_{KJ}	Hz	-5.223
ϕ_j	mHz	7.896
ϕ_{jk}	mHz	-496.365
ϕ_k	mHz	593.908

Table S63: c-$^{13}C^{13}C^{16}O^{16}O$ Geometry [Å or Deg]

Coordinate	r_e	r_0
$r(O_1-C_2)$	1.3813041	1.3890025
$r(O_1-C_3)$	1.3813041	1.3890025
$r(O_1-O_4)$	1.9840284	1.9953246
$\angle(O_1-O_4-C_2)$	44.0962196	44.0891104
$\angle(O_1-O_4-C_3)$	44.0962197	44.0891105

Table S64: c-$^{13}C^{13}C^{16}O^{16}O$ Fermi Resonances

$2\nu_5 = \nu_4$

Table S65: Si(O)OSi Geometry [Å or Deg]

Coordinate	r_e	r_0
$r(O_1-Si_2)$	1.5142983	1.5140618
$r(Si_2-O_3)$	1.7009711	1.7112598
$r(O_3-Si_4)$	1.6660867	1.6670303
$\angle(Si_2-O_1-O_3)$	125.7978085	125.4552259
$\angle(O_3-Si_2-Si_4)$	85.1391323	85.1899607

Table S66: Si(O)OSi Fermi Resonances

$2\nu_3=\nu_5+\nu_2=\nu_1$
$\nu_4+\nu_3=\nu_5+\nu_3=\nu_2$
$2\nu_5=\nu_3$
$2\nu_6=\nu_4$

Table S67: $^{28}Si(^{16}O)^{16}O^{29}Si$ Frequencies and ZPVE

	Description	Frequencies [cm^{-1}]
ω_1	O_1-Si_1 stretch	1246.9
ω_2	O_2-Si_2 stretch	855.4
ω_3	Si_1-O_2 stretch	603.5
ω_4	$Si_1-O_2-Si_2$ bend	440.7
ω_5	$O_1-Si_1-O_2$ bend	206.9
ω_6	out-of-plane bend	166.7
ν_1	O_1-Si_1 stretch	1234.3
ν_2	O_2-Si_2 stretch	853.2
ν_3	Si_1-O_2 stretch	579.9
ν_4	$Si_1-O_2-Si_2$ bend	432.6
ν_5	$O_1-Si_1-O_2$ bend	203.9
ν_6	out-of-plane bend	166.0
ZPVE [cm^{-1}]		1750.9

Table S68: $^{28}Si(^{16}O)^{16}O^{29}Si$ Rotational Constants [MHz]

A_e	26301.4
B_e	3028.8
C_e	2716.1
A_0	26272.2
B_0	3016.0
C_0	2703.2
κ	-0.97346
A_1	26310.1
B_1	3005.0
C_1	2694.9
A_2	26114.2
B_2	3015.3
C_2	2702.0
A_3	26085.5
B_3	3007.4
C_3	2690.0
A_4	26479.8
B_4	3007.3
C_4	2696.9
A_5	26326.1
B_5	3018.4
C_5	2701.4
A_6	26259.0
B_6	3017.0
C_6	2708.6

Table S69: ^{28}Si(^{16}O)^{16}O^{29}Si Quartic and Sextic Distortion Constants

Constant	Units	
Δ_J	Hz	464.156
Δ_K	kHz	205.703
Δ_{JK}	kHz	-1.478
δ_J	Hz	67.442
δ_K	kHz	3.781
Φ_J	uHz	-83.199
Φ_K	Hz	3.651
Φ_{JK}	mHz	14.113
Φ_{KJ}	mHz	-497.433
ϕ_j	uHz	-11.585
ϕ_{jk}	mHz	2.328
ϕ_k	mHz	555.751

Table S70: ^{28}Si(^{16}O)^{16}O^{29}Si Geometry [Å or Deg]

Coordinate	r$_e$	r$_0$
r(O$_1-$Si$_2$)	1.5142983	1.5140371
r(Si$_2-$O$_3$)	1.7009711	1.7112661
r(O$_3-$Si$_4$)	1.6660867	1.6670067
$\angle$(Si$_2-$O$_1-$O$_3$)	125.7978085	125.4553449
$\angle$(O$_3-$Si$_2-$Si$_4$)	85.1391323	85.1896935

Table S71: ^{28}Si(^{16}O)^{16}O^{29}Si Fermi Resonances

$2\nu_3=\nu_5+\nu_2=\nu_1$
$\nu_4+\nu_3=\nu_5+\nu_3=\nu_2$
$2\nu_5=\nu_3$
$2\nu_6=\nu_4$

Table S72: ^{28}Si(^{16}O)^{16}O^{30}Si Frequencies and ZPVE

	Description	Frequencies [cm^{-1}]
ω_1	O$_1-$Si$_1$ stretch	1246.8
ω_2	O$_2-$Si$_2$ stretch	851.6
ω_3	Si$_1-$O$_2$ stretch	602.5
ω_4	Si$_1-$O$_2-$Si$_2$ bend	437.7
ω_5	O$_1-$Si$_1-$O$_2$ bend	206.3
ω_6	out-of-plane bend	166.4
ν_1	O$_1-$Si$_1$ stretch	1234.1
ν_2	O$_2-$Si$_2$ stretch	849.8
ν_3	Si$_1-$O$_2$ stretch	578.9
ν_4	Si$_1-$O$_2-$Si$_2$ bend	429.8
ν_5	O$_1-$Si$_1-$O$_2$ bend	203.3
ν_6	out-of-plane bend	165.7
ZPVE [cm^{-1}]		1746.5

Table S73: ^{28}Si(^{16}O)^{16}O^{30}Si Rotational Constants [MHz]

A_e	26192.0
B_e	2984.1
C_e	2678.9
A_0	26164.1
B_0	2971.5
C_0	2666.3
κ	-0.97402
A_1	26203.0
B_1	2960.7
C_1	2658.1
A_2	26009.5
B_2	2970.7
C_2	2665.0
A_3	25975.0
B_3	2963.2
C_3	2653.5
A_4	26372.1
B_4	2963.0
C_4	2660.1
A_5	26219.2
B_5	2973.8
C_5	2664.5
A_6	26150.4
B_6	2972.5
C_6	2671.6

Table S74: ^{28}Si(^{16}O)^{16}O^{30}Si Quartic and Sextic Distortion Constants

Constant	Units	
Δ_J	Hz	453.245
Δ_K	kHz	206.769
Δ_{JK}	kHz	-1.672
δ_J	Hz	65.542
δ_K	kHz	3.673
Φ_J	uHz	-80.282
Φ_K	Hz	3.597
Φ_{JK}	mHz	13.659
Φ_{KJ}	mHz	-485.144
ϕ_j	uHz	-10.947
ϕ_{jk}	mHz	2.173
ϕ_k	mHz	549.076

Table S75: ^{28}Si(^{16}O)^{16}O^{30}Si Geometry [Å or Deg]

Coordinate	r_e	r_0
$r(O_1-Si_2)$	1.5142983	1.5140137
$r(Si_2-O_3)$	1.7009711	1.7112720
$r(O_3-Si_4)$	1.6660867	1.6669844
$\angle(Si_2-O_1-O_3)$	125.7978085	125.4554615
$\angle(O_3-Si_2-Si_4)$	85.1391323	85.1894376

Table S76: ^{28}Si(^{16}O)^{16}O^{30}Si Fermi Resonances

$2\nu_3=\nu_5+\nu_2=\nu_1$
$\nu_4+\nu_3=\nu_5+\nu_3=\nu_2$
$2\nu_5=\nu_3$
$2\nu_6=\nu_4$

Table S77: $^{28}Si(^{16}O)^{18}O^{28}Si$ Frequencies and ZPVE

	Description	Frequencies [cm^{-1}]
ω_1	O_1-Si_1 stretch	1246.7
ω_2	O_2-Si_2 stretch	828.8
ω_3	Si_1-O_2 stretch	580.3
ω_4	$Si_1-O_2-Si_2$ bend	441.0
ω_5	$O_1-Si_1-O_2$ bend	206.4
ω_6	out-of-plane bend	166.9
ν_1	O_1-Si_1 stretch	1232.9
ν_2	O_2-Si_2 stretch	826.7
ν_3	Si_1-O_2 stretch	558.1
ν_4	$Si_1-O_2-Si_2$ bend	432.8
ν_5	$O_1-Si_1-O_2$ bend	203.5
ν_6	out-of-plane bend	166.2
ZPVE [cm^{-1}]		1726.3

Table S78: $^{28}Si(^{16}O)^{18}O^{28}Si$ Rotational Constants [MHz]

A_e	24115.3
B_e	3065.7
C_e	2720.0
A_0	24096.9
B_0	3053.1
C_0	2707.4
κ	-0.96768
A_1	24133.3
B_1	3041.9
C_1	2699.2
A_2	23942.8
B_2	3052.7
C_2	2706.2
A_3	23947.7
B_3	3044.1
C_3	2694.3
A_4	24284.9
B_4	3044.4
C_4	2701.3
A_5	24149.7
B_5	3055.8
C_5	2705.7
A_6	24086.2
B_6	3053.9
C_6	2712.8

Table S79: $^{28}Si(^{16}O)^{18}O^{28}Si$ Quartic and Sextic Distortion Constants

Constant	Units	
Δ_J	Hz	464.875
Δ_K	kHz	173.095
Δ_{JK}	kHz	-0.486
δ_J	Hz	72.651
δ_K	kHz	3.792
Φ_J	uHz	-82.136
Φ_K	Hz	3.040
Φ_{JK}	mHz	13.863
Φ_{KJ}	mHz	-434.923
ϕ_j	uHz	-14.181
ϕ_{jk}	mHz	2.511
ϕ_k	mHz	490.249

Table S80: ^{28}Si(^{16}O)^{18}O^{28}Si Geometry [Å or Deg]

Coordinate	r_e	r_0
$r(O_1-Si_2)$	1.5142983	1.5140582
$r(Si_2-O_3)$	1.7009711	1.7109596
$r(O_3-Si_4)$	1.6660867	1.6669218
$\angle(Si_2-O_1-O_3)$	125.7978085	125.4581576
$\angle(O_3-Si_2-Si_4)$	85.1391323	85.1973788

Table S81: ^{28}Si(^{16}O)^{18}O^{28}Si Fermi Resonances

$2\nu_3=\nu_3+\nu_2=\nu_1$
$\nu_4+\nu_3=\nu_5+\nu_3=\nu_2$
$2\nu_5=\nu_3$
$2\nu_6=\nu_4$

Table S82: ^{28}Si(^{18}O)^{16}O^{28}Si Frequencies and ZPVE

	Description	Frequencies [cm^{-1}]
ω_1	O_1-Si_1 stretch	1207.2
ω_2	O_2-Si_2 stretch	858.3
ω_3	Si_1-O_2 stretch	603.7
ω_4	$Si_1-O_2-Si_2$ bend	437.2
ω_5	$O_1-Si_1-O_2$ bend	203.2
ω_6	out-of-plane bend	163.6
ν_1	O_1-Si_1 stretch	1198.6
ν_2	O_2-Si_2 stretch	855.2
ν_3	Si_1-O_2 stretch	580.2
ν_4	$Si_1-O_2-Si_2$ bend	429.1
ν_5	$O_1-Si_1-O_2$ bend	200.3
ν_6	out-of-plane bend	163.0
ZPVE [cm^{-1}]		1727.8

Table S83: ^{28}Si(^{18}O)^{16}O^{28}Si Rotational Constants [MHz]

A_e	26417.7
B_e	2913.1
C_e	2623.8
A_0	26386.3
B_0	2901.0
C_0	2611.6
κ	-0.97566
A_1	26424.1
B_1	2890.2
C_1	2603.4
A_2	26224.6
B_2	2900.5
C_2	2610.5
A_3	26207.0
B_3	2892.6
C_3	2599.0
A_4	26587.2
B_4	2893.0
C_4	2605.6
A_5	26438.8
B_5	2903.7
C_5	2610.2
A_6	26373.4
B_6	2902.2
C_6	2617.0

Table S84: $^{28}Si(^{18}O)^{16}O^{28}Si$ Quartic and Sextic Distortion Constants

Constant	Units	
Δ_J	Hz	418.418
Δ_K	kHz	204.919
Δ_{JK}	kHz	-1.018
δ_J	Hz	58.072
δ_K	kHz	3.619
Φ_J	uHz	-67.171
Φ_K	Hz	3.608
Φ_{JK}	mHz	13.259
Φ_{KJ}	mHz	-494.018
ϕ_j	uHz	-9.349
ϕ_{jk}	mHz	2.502
ϕ_k	mHz	542.068

Table S85: $^{28}Si(^{18}O)^{16}O^{28}Si$ Geometry [Å or Deg]

Coordinate	r_e	r_0
$r(O_1-Si_2)$	1.5142983	1.5141699
$r(Si_2-O_3)$	1.7009711	1.7111853
$r(O_3-Si_4)$	1.6660867	1.6670135
$\angle(Si_2-O_1-O_3)$	125.7978085	125.4561284
$\angle(O_3-Si_2-Si_4)$	85.1391323	85.1861714

Table S86: $^{28}Si(^{18}O)^{16}O^{28}Si$ Fermi Resonances

$2\nu_3=\nu_5+\nu_2=\nu_1$
$\nu_4+\nu_3=\nu_5+\nu_3=\nu_2$
$2\nu_5=\nu_3$
$2\nu_6=\nu_4$

Table S87: $^{28}Si(^{18}O)^{18}O^{28}Si$ Frequencies and ZPVE

	Description	Frequencies [cm^{-1}]
ω_1	O_1-Si_1 stretch	1206.8
ω_2	O_2-Si_2 stretch	827.6
ω_3	Si_1-O_2 stretch	579.4
ω_4	$Si_1-O_2-Si_2$ bend	434.3
ω_5	$O_1-Si_1-O_2$ bend	202.2
ω_6	out-of-plane bend	163.5
ν_1	O_1-Si_1 stretch	1195.2
ν_2	O_2-Si_2 stretch	824.9
ν_3	Si_1-O_2 stretch	557.4
ν_4	$Si_1-O_2-Si_2$ bend	426.2
ν_5	$O_1-Si_1-O_2$ bend	199.3
ν_6	out-of-plane bend	162.8
ZPVE [cm^{-1}]		1698.4

Table S88: ^{28}Si(^{18}O)^{18}O^{28}Si Rotational Constants [MHz]

A_e	24113.8
B_e	2902.0
C_e	2590.5
A_0	24095.1
B_0	2890.4
C_0	2578.7
κ	-0.97103
A_1	24132.7
B_1	2879.7
C_1	2570.8
A_2	23940.3
B_2	2890.1
C_2	2577.5
A_3	23950.8
B_3	2882.0
C_3	2566.5
A_4	24277.3
B_4	2882.6
C_4	2573.1
A_5	24147.8
B_5	2893.2
C_5	2577.3
A_6	24084.1
B_6	2891.5
C_6	2584.0

Table S89: ^{28}Si(^{18}O)^{18}O^{28}Si Quartic and Sextic Distortion Constants

Constant	Units	
Δ_J	Hz	408.796
Δ_K	kHz	173.490
Δ_{JK}	kHz	-0.279
δ_J	Hz	60.709
δ_K	kHz	3.528
Φ_J	uHz	-63.524
Φ_K	Hz	2.966
Φ_{JK}	mHz	12.610
Φ_{KJ}	mHz	-420.109
ϕ_j	uHz	-10.809
ϕ_{jk}	mHz	2.507
ϕ_k	mHz	473.723

Table S90: ^{28}Si(^{18}O)^{18}O^{28}Si Geometry [Å or Deg]

Coordinate	r$_e$	r$_0$
r(O$_1$−Si$_2$)	1.5142983	1.5141664
r(Si$_2$−O$_3$)	1.7009711	1.7108837
r(O$_3$−Si$_4$)	1.6660867	1.6669062
∠(Si$_2$−O$_1$−O$_3$)	125.7978085	125.4590691
∠(O$_3$−Si$_2$−Si$_4$)	85.1391323	85.1935991

Table S91: ^{28}Si(^{18}O)^{18}O^{28}Si Fermi Resonances

$2\nu_3$=ν_5+ν_2=ν_1
ν_4+ν_3=ν_5+ν_3=ν_2
$2\nu_5$=ν_3
$2\nu_6$=ν_4

Table S92: ^{29}Si(^{16}O)^{16}O^{28}Si Frequencies and ZPVE

	Description	Frequencies [cm^{-1}]
ω_1	O_1-Si_1 stretch	1237.9
ω_2	O_2-Si_2 stretch	858.5
ω_3	Si_1-O_2 stretch	603.0
ω_4	$Si_1-O_2-Si_2$ bend	441.8
ω_5	$O_1-Si_1-O_2$ bend	206.0
ω_6	out-of-plane bend	165.5
ν_1	O_1-Si_1 stretch	1225.8
ν_2	O_2-Si_2 stretch	855.8
ν_3	Si_1-O_2 stretch	579.5
ν_4	$Si_1-O_2-Si_2$ bend	433.7
ν_5	$O_1-Si_1-O_2$ bend	203.1
ν_6	out-of-plane bend	164.9
ZPVE [cm^{-1}]		1747.4

Table S93: ^{29}Si(^{16}O)^{16}O^{28}Si Rotational Constants [MHz]

A_e	26336.2
B_e	3067.8
C_e	2747.6
A_0	26301.5
B_0	3054.7
C_0	2734.6
κ	-0.97284
A_1	26335.3
B_1	3043.7
C_1	2726.3
A_2	26143.4
B_2	3054.2
C_2	2733.5
A_3	26117.3
B_3	3045.7
C_3	2721.0
A_4	26504.4
B_4	3045.9
C_4	2728.1
A_5	26350.1
B_5	3057.3
C_5	2732.7
A_6	26289.3
B_6	3055.5
C_6	2740.0

Table S94: ^{29}Si(^{16}O)^{16}O^{28}Si Quartic and Sextic Distortion Constants

Constant	Units	
Δ_J	Hz	475.674
Δ_K	kHz	199.357
Δ_{JK}	kHz	-1.281
δ_J	Hz	69.319
δ_K	kHz	3.860
Φ_J	uHz	-87.760
Φ_K	Hz	3.636
Φ_{JK}	mHz	14.166
Φ_{KJ}	mHz	-499.502
ϕ_j	uHz	-11.995
ϕ_{jk}	mHz	2.397
ϕ_k	mHz	540.053

Table S95: $^{29}Si(^{16}O)^{16}O^{28}Si$ Geometry [Å or Deg]

Coordinate	r_e	r_0
$r(O_1-Si_2)$	1.5142983	1.5140580
$r(Si_2-O_3)$	1.7009711	1.7111886
$r(O_3-Si_4)$	1.6660867	1.6670751
$\angle(Si_2-O_1-O_3)$	125.7978085	125.4598827
$\angle(O_3-Si_2-Si_4)$	85.1391323	85.1882112

Table S96: $^{29}Si(^{16}O)^{16}O^{28}Si$ Fermi Resonances

$2\nu_3=\nu_5+\nu_2=\nu_1$
$\nu_4+\nu_3=\nu_5+\nu_3=\nu_2$
$2\nu_5=\nu_3$
$2\nu_6=\nu_4$

Table S97: $^{30}Si(^{16}O)^{16}O^{28}Si$ Frequencies and ZPVE

	Description	Frequencies [cm^{-1}]
ω_1	O_1-Si_1 stretch	1229.3
ω_2	O_2-Si_2 stretch	857.6
ω_3	Si_1-O_2 stretch	601.5
ω_4	$Si_1-O_2-Si_2$ bend	440.0
ω_5	$O_1-Si_1-O_2$ bend	204.7
ω_6	out-of-plane bend	164.1
ν_1	O_1-Si_1 stretch	1217.6
ν_2	O_2-Si_2 stretch	854.7
ν_3	Si_1-O_2 stretch	578.2
ν_4	$Si_1-O_2-Si_2$ bend	431.9
ν_5	$O_1-Si_1-O_2$ bend	201.8
ν_6	out-of-plane bend	163.6
ZPVE [cm^{-1}]		1739.8

Table S98: $^{30}Si(^{16}O)^{16}O^{28}Si$ Rotational Constants [MHz]

A_e	26256.7
B_e	3059.7
C_e	2740.4
A_0	26218.5
B_0	3046.7
C_0	2727.3
κ	-0.97281
A_1	26249.4
B_1	3036.0
C_1	2719.2
A_2	26063.7
B_2	3046.4
C_2	2726.4
A_3	26034.0
B_3	3037.6
C_3	2713.7
A_4	26417.5
B_4	3037.9
C_4	2720.8
A_5	26263.3
B_5	3049.2
C_5	2725.4
A_6	26206.7
B_6	3047.4
C_6	2732.7

Table S99: $^{30}Si(^{16}O)^{16}O^{28}Si$ Quartic and Sextic Distortion Constants

Constant	Units	
Δ_J	Hz	475.572
Δ_K	kHz	194.545
Δ_{JK}	kHz	-1.292
δ_J	Hz	69.171
δ_K	kHz	3.823
Φ_J	uHz	-89.214
Φ_K	Hz	3.574
Φ_{JK}	mHz	13.751
Φ_{KJ}	mHz	-488.989
ϕ_j	uHz	-11.752
ϕ_{jk}	mHz	2.300
ϕ_k	mHz	518.869

Table S100: $^{30}Si(^{16}O)^{16}O^{28}Si$ Geometry [Å or Deg]

Coordinate	r_e	r_0
$r(O_1-Si_2)$	1.5142983	1.5140543
$r(Si_2-O_3)$	1.7009711	1.7111213
$r(O_3-Si_4)$	1.6660867	1.6671173
$\angle(Si_2-O_1-O_3)$	125.7978085	125.4642794
$\angle(O_3-Si_2-Si_4)$	85.1391323	85.1865688

Table S101: $^{30}Si(^{16}O)^{16}O^{28}Si$ Fermi Resonances

$2\nu_3=\nu_5+\nu_2=\nu_1$
$\nu_4+\nu_3=\nu_5+\nu_3=\nu_2$
$2\nu_5=\nu_3$
$2\nu_6=\nu_4$

Table S102: c-Si_2O_2 Geometry [Å or Deg]

Coordinate	r_e	r_0
$r(O_1-Si_2)$	1.6867196	1.6915087
$r(O_1-Si_3)$	1.6867196	1.6915087
$r(O_1-O_4)$	2.3335636	2.3411308
$\angle(O_1-O_4-Si_2)$	46.2315078	46.2093635
$\angle(O_1-O_4-Si_3)$	46.2315078	46.2093635

Table S103: c-Si_2O_2 Fermi Resonances

$2\nu_6=\nu_5$

Table S104: c-$^{28}Si^{28}Si^{16}O^{18}O$ Frequencies and ZPVE

	Description	Frequencies [cm^{-1}]
ω_1	(O_1-O_2 stretch) + (Si_1-Si_2 stretch)	846.4
ω_2	(O_1-Si_1 stretch) - (O_1-Si_2 stretch) + (Si_1-O_2 stretch) - (Si_2-O_2 stretch)	813.9
ω_3	(O_1-Si_1 stretch) + (O_1-Si_2 stretch) - (Si_1-O_2 stretch) - (Si_2-O_2 stretch)	768.3
ω_4	(O_1-Si_1 stretch) - (O_1-Si_2 stretch) - (Si_1-O_2 stretch) + (Si_2-O_2 stretch)	566.6
ω_5	(O_1-O_2 stretch) - (Si_1-Si_2 stretch)	556.9
ω_6	out-of-plane bend	233.1
ν_1	(O_1-O_2 stretch) + (Si_1-Si_2 stretch)	832.8
ν_2	(O_1-Si_1 stretch) - (O_1-Si_2 stretch) + (Si_1-O_2 stretch) - (Si_2-O_2 stretch)	800.6
ν_3	(O_1-Si_1 stretch) + (O_1-Si_2 stretch) - (Si_1-O_2 stretch) - (Si_2-O_2 stretch)	757.4
ν_4	(O_1-Si_1 stretch) - (O_1-Si_2 stretch) - (Si_1-O_2 stretch) + (Si_2-O_2 stretch)	553.4
ν_5	(O_1-O_2 stretch) - (Si_1-Si_2 stretch)	553.8
ν_6	out-of-plane bend	236.5
ZPVE [cm^{-1}]		1887.6

Table S105: c-$^{28}Si^{28}Si^{16}O^{18}O$ Rotational Constants [MHz]

A_e	10934.6
B_e	6087.9
C_e	3910.5
A_0	10882.5
B_0	6066.3
C_0	3891.4
κ	-0.37782
A_1	10859.2
B_1	6056.6
C_1	3888.5
A_2	10844.7
B_2	6064.4
C_2	3877.8
A_3	10897.0
B_3	6047.0
C_3	3881.1
A_4	10855.1
B_4	6051.5
C_4	3875.3
A_5	10886.0
B_5	6071.0
C_5	3889.1
A_6	10848.9
B_6	6064.3
C_6	3898.4

Table S106: c-$^{28}Si^{28}Si^{16}O^{18}O$ Quartic and Sextic Distortion Constants

Constant	Units	
Δ_J	kHz	1.602
Δ_K	kHz	7.412
Δ_{JK}	kHz	1.979
δ_J	Hz	578.205
δ_K	kHz	3.697
Φ_J	mHz	1.316
Φ_K	mHz	50.480
Φ_{JK}	mHz	-8.078
Φ_{KJ}	mHz	-24.066
ϕ_j	uHz	651.299
ϕ_{jk}	mHz	-1.699
ϕ_k	mHz	29.735

Table S107: c-$^{28}Si^{28}Si^{16}O^{18}O$ Geometry [Å or Deg]

Coordinate	r_e	r_0
$r(O_1{-}Si_2)$	1.6867196	1.6914498
$r(O_1{-}Si_3)$	1.6867196	1.6914498
$r(O_1{-}O_4)$	2.3335636	2.3409727
$\angle(O_1{-}O_4{-}Si_2)$	46.2315078	46.2093800
$\angle(O_1{-}O_4{-}Si_3)$	46.2315078	46.2093800

Table S108: c-$^{28}Si^{28}Si^{16}O^{18}O$ Fermi Resonances

$2\nu_6{=}\nu_5$

Table S109: c-$^{28}Si^{28}Si^{18}O^{18}O$ Frequencies and ZPVE

	Description	Frequencies [cm^{-1}]
ω_1	(O_1-O_2 stretch) + (Si_1-Si_2 stretch)	824.7
ω_2	(O_1-Si_1 stretch) - (O_1-Si_2 stretch) + (Si_1-O_2 stretch) - (Si_2-O_2 stretch)	798.2
ω_3	(O_1-Si_1 stretch) + (O_1-Si_2 stretch) - (Si_1-O_2 stretch) - (Si_2-O_2 stretch)	757.4
ω_4	(O_1-Si_1 stretch) - (O_1-Si_2 stretch) - (Si_1-O_2 stretch) + (Si_2-O_2 stretch)	556.5
ω_5	(O_1-O_2 stretch) - (Si_1-Si_2 stretch)	552.5
ω_6	out-of-plane bend	228.8
ν_1	(O_1-O_2 stretch) + (Si_1-Si_2 stretch)	811.7
ν_2	(O_1-Si_1 stretch) - (O_1-Si_2 stretch) + (Si_1-O_2 stretch) - (Si_2-O_2 stretch)	785.6
ν_3	(O_1-Si_1 stretch) + (O_1-Si_2 stretch) - (Si_1-O_2 stretch) - (Si_2-O_2 stretch)	746.8
ν_4	(O_1-Si_1 stretch) - (O_1-Si_2 stretch) - (Si_1-O_2 stretch) + (Si_2-O_2 stretch)	543.8
ν_5	(O_1-O_2 stretch) - (Si_1-Si_2 stretch)	549.5
ν_6	out-of-plane bend	232.0
ZPVE [cm^{-1}]		1854.3

Table S110: c-$^{28}Si^{28}Si^{18}O^{18}O$ Rotational Constants [MHz]

A_e	10312.3
B_e	6087.9
C_e	3828.0
A_0	10264.2
B_0	6066.7
C_0	3809.4
κ	-0.30060
A_1	10240.4
B_1	6057.4
C_1	3802.9
A_2	10228.9
B_2	6065.3
C_2	3799.9
A_3	10279.1
B_3	6047.2
C_3	3799.4
A_4	10239.2
B_4	6052.2
C_4	3794.1
A_5	10267.4
B_5	6071.5
C_5	3807.1
A_6	10234.0
B_6	6064.2
C_6	3816.1

Table S111: c-$^{28}Si^{28}Si^{18}O^{18}O$ Quartic and Sextic Distortion Constants

Constant	Units	
Δ_J	kHz	1.589
Δ_K	kHz	6.382
Δ_{JK}	kHz	1.788
δ_J	Hz	585.110
δ_K	kHz	3.420
Φ_J	mHz	1.314
Φ_K	mHz	43.270
Φ_{JK}	mHz	-8.166
Φ_{KJ}	mHz	-19.999
ϕ_j	uHz	652.029
ϕ_{jk}	mHz	-1.904
ϕ_k	mHz	24.583

Table S112: c-$^{28}Si^{28}Si^{18}O^{18}O$ Geometry [Å or Deg]

Coordinate	r_e	r_0
$r(O_1-Si_2)$	1.6867196	1.6913413
$r(O_1-Si_3)$	1.6867196	1.6913413
$r(O_1-O_4)$	2.3335636	2.3408221
$\angle(O_1-O_4-Si_2)$	46.2315078	46.2111774
$\angle(O_1-O_4-Si_3)$	46.2315078	46.2111774

Table S113: c-$^{28}Si^{28}Si^{18}O^{18}O$ Fermi Resonances

$2\nu_6=\nu_5$

Table S114: c-$^{28}Si^{29}Si^{16}O^{16}O$ Frequencies and ZPVE

	Description	Frequencies [cm^{-1}]
ω_1	(O_1-O_2 stretch) + (Si_1-Si_2 stretch)	859.8
ω_2	(O_1-Si_1 stretch) - (O_1-Si_2 stretch) + (Si_1-O_2 stretch) - (Si_2-O_2 stretch)	825.4
ω_3	(O_1-Si_1 stretch) + (O_1-Si_2 stretch) - (Si_1-O_2 stretch) - (Si_2-O_2 stretch)	783.3
ω_4	(O_1-Si_1 stretch) - (O_1-Si_2 stretch) - (Si_1-O_2 stretch) + (Si_2-O_2 stretch)	576.3
ω_5	(O_1-O_2 stretch) - (Si_1-Si_2 stretch)	557.4
ω_6	out-of-plane bend	236.6
ν_1	(O_1-O_2 stretch) + (Si_1-Si_2 stretch)	845.9
ν_2	(O_1-Si_1 stretch) - (O_1-Si_2 stretch) + (Si_1-O_2 stretch) - (Si_2-O_2 stretch)	811.8
ν_3	(O_1-Si_1 stretch) + (O_1-Si_2 stretch) - (Si_1-O_2 stretch) - (Si_2-O_2 stretch)	772.1
ν_4	(O_1-Si_1 stretch) - (O_1-Si_2 stretch) - (Si_1-O_2 stretch) + (Si_2-O_2 stretch)	562.6
ν_5	(O_1-O_2 stretch) - (Si_1-Si_2 stretch)	554.4
ν_6	out-of-plane bend	240.1
ZPVE [cm^{-1}]		1914.3

Table S115: c-$^{28}Si^{29}Si^{16}O^{16}O$ Rotational Constants [MHz]

A_e	11604.4
B_e	5982.1
C_e	3947.4
A_0	11547.9
B_0	5960.7
C_0	3927.8
κ	-0.46643
A_1	11520.4
B_1	5951.9
C_1	3921.6
A_2	11506.9
B_2	5958.4
C_2	3917.8
A_3	11567.1
B_3	5940.9
C_3	3916.8
A_4	11518.3
B_4	5945.7
C_4	3910.9
A_5	11551.2
B_5	5965.1
C_5	3925.5
A_6	11510.1
B_6	5959.4
C_6	3935.0

Table S116: c-$^{28}Si^{29}Si^{16}O^{16}O$ Quartic and Sextic Distortion Constants

Constant	Units	
Δ_J	kHz	1.566
Δ_K	kHz	8.675
Δ_{JK}	kHz	2.117
δ_J	Hz	549.056
δ_K	kHz	3.925
Φ_J	mHz	1.251
Φ_K	mHz	57.776
Φ_{JK}	mHz	-7.622
Φ_{KJ}	mHz	-28.007
ϕ_j	uHz	616.809
ϕ_{jk}	mHz	-1.368
ϕ_k	mHz	35.950

Table S117: c-$^{28}Si^{29}Si^{16}O^{16}O$ Geometry [Å or Deg]

Coordinate	r_e	r_0
r(O_1−Si_2)	1.6867196	1.6914975
r(O_1−Si_3)	1.6867196	1.6914876
r(O_1−O_4)	2.3335636	2.3411121
∠(O_1−O_4−Si_2)	46.2315078	46.2094396
∠(O_1−O_4−Si_3)	46.2315078	46.2091193

Table S118: c-$^{28}Si^{29}Si^{16}O^{16}O$ Fermi Resonances

$2\nu_6 = \nu_5$

Table S119: c-$^{28}Si^{30}Si^{16}O^{16}O$ Frequencies and ZPVE

	Description	Frequencies [cm^{-1}]
ω_1	(O_1−O_2 stretch) + (Si_1−Si_2 stretch)	858.5
ω_2	(O_1−Si_1 stretch) - (O_1−Si_2 stretch) + (Si_1−O_2 stretch) - (Si_2−O_2 stretch)	822.7
ω_3	(O_1−Si_1 stretch) + (O_1−Si_2 stretch) - (Si_1−O_2 stretch) - (Si_2−O_2 stretch)	781.0
ω_4	(O_1−Si_1 stretch) - (O_1−Si_2 stretch) - (Si_1−O_2 stretch) + (Si_2−O_2 stretch)	574.6
ω_5	(O_1−O_2 stretch) - (Si_1−Si_2 stretch)	553.7
ω_6	out-of-plane bend	235.9
ν_1	(O_1−O_2 stretch) + (Si_1−Si_2 stretch)	844.7
ν_2	(O_1−Si_1 stretch) - (O_1−Si_2 stretch) + (Si_1−O_2 stretch) - (Si_2−O_2 stretch)	809.2
ν_3	(O_1−Si_1 stretch) + (O_1−Si_2 stretch) - (Si_1−O_2 stretch) - (Si_2−O_2 stretch)	769.9
ν_4	(O_1−Si_1 stretch) - (O_1−Si_2 stretch) - (Si_1−O_2 stretch) + (Si_2−O_2 stretch)	561.0
ν_5	(O_1−O_2 stretch) - (Si_1−Si_2 stretch)	550.7
ν_6	out-of-plane bend	239.3
ZPVE [cm^{-1}]		1908.2

Table S120: c-$^{28}Si^{30}Si^{16}O^{16}O$ Rotational Constants [MHz]

A_e	11604.4
B_e	5882.5
C_e	3903.6
A_0	11548.0
B_0	5861.5
C_0	3884.5
κ	-0.48403
A_1	11520.4
B_1	5853.1
C_1	3878.7
A_2	11507.2
B_2	5859.1
C_2	3874.7
A_3	11567.4
B_3	5842.2
C_3	3873.4
A_4	11518.5
B_4	5846.8
C_4	3867.8
A_5	11551.2
B_5	5865.8
C_5	3882.3
A_6	11510.0
B_6	5860.4
C_6	3891.6

Table S121: c-$^{28}Si^{30}Si^{16}O^{16}O$ Quartic and Sextic Distortion Constants

Constant	Units	
Δ_J	kHz	1.519
Δ_K	kHz	8.751
Δ_{JK}	kHz	2.088
δ_J	Hz	528.926
δ_K	kHz	3.872
Φ_J	mHz	1.190
Φ_K	mHz	57.262
Φ_{JK}	mHz	-7.246
Φ_{KJ}	mHz	-27.808
ϕ_j	uHz	586.369
ϕ_{jk}	mHz	-1.249
ϕ_k	mHz	36.010

Table S122: c-$^{28}Si^{30}Si^{16}O^{16}O$ Geometry [Å or Deg]

Coordinate	r_e	r_0
$r(O_1-Si_2)$	1.6867196	1.6914868
$r(O_1-Si_3)$	1.6867196	1.6914677
$r(O_1-O_4)$	2.3335636	2.3410944
$\angle(O_1-O_4-Si_2)$	46.2315078	46.2095086
$\angle(O_1-O_4-Si_3)$	46.2315078	46.2088869

Table S123: c-$^{28}Si^{30}Si^{16}O^{16}O$ Fermi Resonances

$2\nu_6=\nu_5$
